# Comparative Mitogenomics Reveals Gene Rearrangement and Phylogenetic Relationships in Siphlonuroidea (Insecta: Ephemeroptera)

**DOI:** 10.3390/insects17070718

**Published:** 2026-07-11

**Authors:** Chen-Xiao Hu, Ting Yang, Hui-Yuan Wu, Yi-Jie Lin, Yi-Xin Gao, Ya-Jie Gao, Dan-Na Yu, Jia-Yong Zhang

**Affiliations:** 1 College of Life Sciences, Zhejiang Normal University, Jinhua 321004, China; hcx1124@zjnu.edu.cn (C.-X.H.); 2731849139.yt@zjnu.edu.cn (T.Y.); 202031880407@zjnu.edu.cn (H.-Y.W.); inkallen@zjnu.edu.cn (Y.-X.G.); ydn@zjnu.cn (D.-N.Y.); 2 MOE Frontier Science Center for Brain Science and Brain-Machine Integration, State Key Laboratory of Brain-Machine Intelligence, Zhejiang University, Hangzhou 311121, China; 12318270@zju.edu.cn; 3 School of Biological Engineering, Aksu Vocational and Technical College, Aksu 843100, China; 4 Key Lab of Wildlife Biotechnology, Conservation and Utilization of Zhejiang Province, Zhejiang Normal University, Jinhua 321004, China

**Keywords:** Ephemeroptera, mitogenomics, mitochondrial gene order, molecular phylogeny, divergence time estimation, evolutionary history

## Abstract

Siphlonuroidea is a superfamily within Ephemeroptera, yet the phylogenetic relationships among its constituent families and their placement relative to other mayfly lineages remain unresolved. To address these questions, we generated and analyzed 16 newly assembled mitochondrial genomes from 14 species across Ephemeroptera, including three species of Ameletidae, four of Siphlonuridae, and nine mitochondrial genomes from seven species of Isonychiidae. Comparative mitogenomic analysis revealed two distinct tRNA gene rearrangement patterns within Siphlonuridae, including *trnI-trnQ-trnM-trnQ-trnM-trnQ-trnM-trnQ-trnM* and *trnI-trnM-trnQ-trnM*. In contrast, all Ameletidae mitogenomes share an identical rearrangement of *trnI-trnQ-trnM-trnM,* which constitutes a potential synapomorphy supporting the monophyly of this family. Compositional analysis further showed that Siphlonuridae and Ameletidae exhibit significantly higher and highly similar A+T contents, clustering together in hierarchical analyses. This characteristic contrasts sharply with Isonychiidae, which displays markedly lower A+T content. Phylogenomic inference based on the PCG12 dataset supports a sister-group relationship between Siphlonuridae and Ameletidae, with this clade itself forming the sister group to a well-supported clade of Isonychiidae and Heptageniidae. Divergence time estimation places the origin of the Ameletidae and Siphlonuridae lineage in the Late Jurassic (174.71 Mya), while Isonychiidae diverged in the Early Cretaceous (136.81 Mya). In conclusion, Siphlonuridae and Ameletidae show a closer affinity and belong to Siphlonuroidea. Isonychiidae shares a closer relationship with Heptageniidae and remains outside Siphlonuroidea. Siphluriscidae is recovered as the sister lineage to all other extant Ephemeroptera, confirming its status as the earliest-diverging extant mayfly lineage.

## Simple Summary

Mayflies are a pivotal lineage for reconstructing the evolutionary history of insects. The systematic placement and phylogenetic relationships within the mayfly superfamily Siphlonuroidea have remained contentious despite decades of morphological and molecular investigation. To explore these uncertainties, we sequenced 16 newly assembled mitochondrial genomes representing key lineages across Ephemeroptera. Phylogenomic analyses support Siphluriscidae as the sister group to all other extant mayflies and reveal a close phylogenetic affinity between Isonychiidae and Heptageniidae. Furthermore, the results indicate that the traditional circumscription of Siphlonuroidea, particularly its inclusion of Isonychiidae, is not monophyletic, whereas Ameletidae and Siphlonuridae belong within this superfamily.

## 1. Introduction

The superfamily Siphlonuroidea is a relatively small lineage within Ephemeroptera, comprising nymphs characterized by large body size, streamlined morphology, and good swimming proficiency [1,2]. Its constituent families exhibit pronounced external morphological disparity and are predominantly distributed across the Northern Hemisphere [1]. The taxonomic history of Siphlonuroidea spans over 150 years. Eaton established the genus *Siphlonurus* and designated *Siphlonurus flavidus* as the type species [3], providing the nomenclatural foundation for subsequent authors to formally establish the superfamily Siphlonuroidea. Ulmer elevated *Siphlonurus* to familial rank as Siphlonuridae, incorporating both *Siphlonurus* and *Ameletus* [4]. Based on morphological evidence, Kluge et al. proposed the circumscription of Siphlonuroidea, discussed its phylogenetic position, and defined its constituent families [1]. Siphlonuroidea consist of a Northern Hemisphere group of families (Siphlonuridae s. str., Dipteromimidae, Ameletidae, Metretopodidae, Acanthametropodidae and Ametropodidae) and a Southern Hemisphere group of families (Oniscigastridae, Nesameletidae, Rallidentidae, and Ameletopsidae). This classification framework achieved wide acceptance in subsequent research. This also implies that the traditional Siphlonuroidea, which previously included Isonychiidae [5,6], may be non-monophyletic, meaning that the monophyly of this superfamily remains a subject for further discussion.

Traditional morphological classifications frequently placed Isonychiidae within Siphlonuroidea. Tshernova assigned the group to Siphlonuroidea based on morphological characteristics [5]. Similarly, Edmunds placed the Isonychiinae within Siphlonuridae [6]. However, molecular phylogenetic studies often suggest a closer relationship between Isonychiidae and Heptageniidae. Hebert et al. recovered Isonychiidae as sister to Heptageniidae using the *COX1* gene [7]. Ogden et al. resolved Isonychiidae and Heptageniidae as sister lineages using large-scale genomic data [8]. These differing perspectives leave some uncertainties regarding the phylogenetic position of the family. Therefore, expanded taxon sampling combined with mitochondrial genomic analysis may help provide some complementary reference evidence for understanding its systematic status.

The family Siphluriscidae was also briefly accommodated within the superfamily Siphlonuroidea [9,10,11]. Ulmer originally described the genus *Siphluriscus*, designating *Siphluriscus chinensis* Ulmer, 1920 as its type species and provisionally assigned it to Siphlonuridae [9]. Edmunds and Koss transferred *Siphluriscus* to the subfamily Acanthametropodinae, a group included within Siphlonuroidea [10]. Zhou and Peters comprehensively described the morphology of the nymph, female imago and egg of *S. chinensis*, establishing Siphluriscidae fam. nov. within Siphlonuroidea based on imaginal and nymphal characteristics [11]. However, accumulating evidence indicates that Siphluriscidae should be excluded from the traditional Siphlonuroidea, as it constitutes an independent branch at the basal position of all extant Ephemeroptera. Molecular phylogenetic investigations have validated this taxonomic placement through multi-locus [12], mitogenomic [13,14], and phylogenomic approaches [8], resolving Siphluriscidae as the earliest-diverging lineage within Ephemeroptera.

The evolutionary origin of Ephemeroptera extends to the Late Carboniferous or Early Permian, with a major phase of adaptive radiation occurring during the Cretaceous [15,16,17]. Molecular clock analyses have yielded increasingly refined estimates for the divergence of extant lineages. Misof et al. inferred the crown-group origin of Ephemeroptera in the Early Jurassic (~178.82 Mya) [18]. García-Girón et al. pushed this estimate further back to the Late Triassic (~225.14 Mya) [19]. Tong et al. applied four fossil calibration points to date 14 mayfly families, recovering Siphluriscidae and Isonychiidae as diverging in the Early Jurassic (196.91 Mya and 187.82 Mya, respectively), while the Siphlonuridae + Ameletidae lineage originated in the Late Jurassic (~159.99 Mya) [20]. Guo et al. reached similar conclusions using five fossil calibration points [21]. Research regarding the origin and divergence times of individual families within Ephemeroptera remains relatively limited. Therefore, further analyses may help provide new insights into the divergence times of individual families within Ephemeroptera.

Mitochondrial DNA (mtDNA) is a cornerstone molecular marker for phylogenetic inference and cryptic species delimitation, owing to its relatively rapid evolutionary rate, maternal inheritance, and compact, haploid genome structure [13,22,23,24,25,26,27,28,29,30,31,32]. Complete mitogenomes of Ephemeroptera typically span 14–20 kb and encode the canonical metazoan set: 13 protein-coding genes (PCGs), 22 transfer RNA genes (tRNA), two ribosomal RNA genes (rRNA), and a non-coding control region (CR) [20,21,22,33,34]. While mitochondrial gene order is highly conserved across most insect orders, mayflies exhibit notable structural plasticity [14,21,35,36,37]. Mitochondrial gene rearrangements may frequently serve as potential evolutionary markers for exploring phylogenetic relationships across various insect groups [38,39]. However, certain lineages might occasionally exhibit rare and complex instances of homoplasy or intraspecific variation [40].

To further investigate the long-standing phylogenetic uncertainties surrounding the internal relationships of Siphlonuroidea and its placement within Ephemeroptera, we generated 16 new mitogenomes representing 14 distinct species. The dataset includes three species from Siphlonuridae, four from Ameletidae, and seven from Isonychiidae. Among these species, 11 have their mitochondrial genomes deposited in NCBI for the first time, further enriching the mitogenomic resources of Ephemeroptera. By integrating these new data with publicly available mitogenomic sequences from NCBI, this study addresses three tightly integrated objectives: (1) analyzing the gene structural characteristics and gene rearrangement patterns of mitogenomes across families; (2) reconstructing the phylogenetic tree of Ephemeroptera to investigate the phylogenetic relationships of Isonychiidae, Siphlonuridae, and Ameletidae, thereby reassessing the systematic position of Siphlonuroidea; and (3) constructing divergence times using a fossil-calibrated molecular clock with five calibration points.

## 2. Materials and Methods

### 2.1. Sample Collection and Morphological Identification

Between 2023 and 2025, 16 larval specimens of mayfly were collected using standard D-frame kick nets from lotic habitats. The samples represent 14 species spanning three families as detailed in Table 1. Fifteen specimens were collected from 12 geographically distinct localities across eight provinces in China. The remaining specimen was collected from the Rideau River, Ottawa, Canada. All specimens were taxonomically identified to species level based on diagnostic morphological characteristics of the head capsule (including antennae, labrum, mandibles, maxillae, labium, and hypopharynx), thoracic legs, and terminal abdominal structures (cerci). Six specimens correspond to undescribed taxa (Table 1), following standard nomenclatural practice for provisional species assignments. Identification was conducted under an Olympus SZX16 stereomicroscope (Olympus Corporation, Tokyo, Japan), with voucher images and morphological notes archived digitally. All specimens were stored at −20 °C in Zhang’s laboratory, College of Life Sciences, Zhejiang Normal University.

### 2.2. DNA Extraction and Mitogenome Sequencing

Total genomic DNA was extracted from thoracic muscle tissue using the Ezup Column Animal Genomic DNA Purification Kit (Sangon Biotech Company, Shanghai, China), following the manufacturer’s protocol. The mitochondrial *COI* gene fragment was amplified via PCR using the universal primers LCO1490: 5′-GGTCAACAAATCATAAAGATATTGG-3′ and HCO2198: 5′-TAAACTTCAGGGTGACCAAAAAATCA-3′ [41]. Amplified products were purified and Sanger-sequenced by Hangzhou Youkang Biotechnology Co., Ltd. (Hangzhou, China). Species identity was verified by conducting online BLASTn alignment of *COI* sequences against the NCBI Nucleotide database; all molecular identifications were congruent with independent morphological diagnoses. For mitogenome sequencing, high-molecular-weight DNA was subjected to paired-end library preparation and sequenced on the Illumina HiSeq 2000 platform (BerryGenomics, Beijing, China). Raw reads were trimmed and filtered for adapter contamination and low-quality bases using fastp 1.0.1 [42]. De novo assembly of complete mitochondrial genomes was performed independently using three complementary tools: GetOrganelle v.1.7.1 [43], NOVOPlasty v.4.2 [44], and MitoZ [45]. Consensus mitogenome sequences were generated by integrating and manually curating the outputs of all three assemblers, followed by rigorous annotation validation.

### 2.3. Mitogenome Assembling, Annotation and Structural Analysis

The annotation of tRNA genes was performed using the MITOS2 [46] service in Galaxy (https://usegalaxy.eu, accessed on 1 February 2026). For the 13 PCGs and 2 rRNAs, the mitogenomes with the highest similarity were downloaded from the NCBI database as references. These genes were annotated by aligning homologous domains with the closest reference sequences using ClustalW in MEGA v.11 [47] based on the invertebrate mitochondrial genetic code, ensuring that all PCGs were translated accurately. To visually represent the genomic characteristics, circular mitogenome maps were generated using the Proksee [48] (https://proksee.ca, accessed on 2 February 2026), and tRNA secondary structures were visualized using Forna [49] (http://rna.tbi.univie.ac.at/forna/, accessed on 4 February 2026). PhyloSuite v.1.2.3 [50] was utilized to analyze amino acid usage frequencies, relative synonymous codon usage (RSCU), and nucleotide composition bias. The nucleotide skewness was calculated using the following formulas: A-T skew = (A − T)/(A + T), G-C skew = (G − C)/(G + C) [51].

### 2.4. Dataset Selection and Phylogenetic Analyses

To explore the phylogenetic relationships among Siphluriscidae, Siphlonuridae, Ameletidae, and Isonychiidae within Ephemeroptera, we constructed a mitogenomic dataset comprising 164 taxa: 16 newly sequenced mitogenomes (Table 1) and 148 publicly available sequences retrieved from the NCBI database (Table S1). The latter encompassed 112 species representing the only 18 Ephemeroptera families with mitochondrial genome sequences currently available on NCBI [13,14,20,21,33,34,35,36,37,52,53,54,55,56,57,58,59,60,61,62,63,64,65,66,67,68,69,70], as well as six Archaeognatha [71,72,73,74,75], four Zygentoma [76,77,78], 10 Odonata [52,62,79,80,81,82,83,84,85], and 16 Neoptera [86,87,88,89,90,91,92,93] species. Six Archaeognatha sequences were designated as the primary outgroup based on their phylogenetic position as the sister lineage to Dicondylia.

The 13 PCGs were extracted via PhyloSuite v.1.2.3 [50] and aligned using MAFFT v.7.505 [94]. Poorly aligned and hypervariable regions were trimmed using Gblocks v.0.91b [95]. Aligned PCG partitions were concatenated into a single supermatrix. Substitution saturation across codon positions was assessed using DAMBE [96]. Due to the saturation observed at the third position, these positions were excluded to mitigate the potential impact of base compositional bias and high substitution rates [22,97]. Only the first and second codon positions (PCG12) were utilized in subsequent analyses (Table S2). Phylogenetic trees were constructed using Bayesian inference (BI) and maximum likelihood (ML). The BI tree was constructed using MrBayes v.3.2.7a [98] based on the optimal partitioning scheme and evolutionary models from PartitionFinder v.2.1.1 [99] (Table S3). The analysis was run for 10 million generations with sampling every 1000 generations. The first 25% of the samples were discarded as burn-in. Convergence was confirmed when the average standard deviation of split frequencies fell below 0.01, and effective sample sizes (ESS) exceeded 200 for all parameters. The ML tree was generated in IQ-TREE v.3.0.1 [100]. ModelFinder [101] selected the best partitioning and substitution models (Table S4). Node support was assessed via the ultrafast bootstrap method [102]. Final tree visualizations were generated in FigTree v.1.4.4 [103] and refined using ChiPlot [104]. Final figures were polished in Affinity Designer v.3.2.1 [105].

### 2.5. Divergence Time Estimation

Divergence time across Ephemeroptera was estimated using MCMCTree implemented in the PAML v.4.10.7 package [106]. Five fossil calibration points [107,108,109,110] selected for strong phylogenetic placement and stratigraphic reliability were integrated from the primary literature and the Fossilworks database (www.fossilworks.org, accessed on 6 February 2026) (Table 2). The root age constraint was set to 435 Mya, corresponding to the well-supported divergence between Archaeognatha and Zygentoma as reported in the TimeTree 5 database (https://timetree.org/, accessed on 6 February 2026). The baseml program was used to calculate nucleotide substitution rates. Approximate likelihood analysis was performed in MCMCTree with the following parameters: usedata = 3, RootAge < 4.35, burnin = 2,000,000, sampfreq = 100, and nsample = 100,000. MCMC convergence was assessed using Tracer v.1.7.2 [111]. MCMC convergence was confirmed when all parameters reached an effective sample size (ESS) > 200, and trace plots showed adequate mixing and stationarity. The final chronogram was visualized using FigTree v.1.4.4 [103] and ChiPlot [104], followed by aesthetic polishing in Affinity Designer v.3.2.1 [105].

## 3. Results

### 3.1. Mitogenomes Structure Analysis

**Figure 1:**
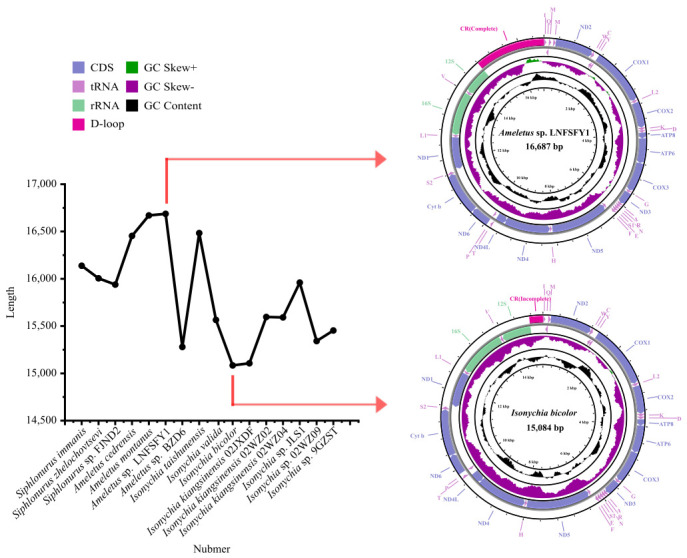
Line plot of the lengths of newly obtained mitogenomes.

This study obtained 16 newly assembled mitogenomes from three families of Ephemeroptera: Siphlonuridae (*n* = 3), Ameletidae (*n* = 4), and Isonychiidae (*n* = 9). Genome lengths ranged from 15,084 bp (*Isonychia bicolor*) to 16,687 bp (*Ameletus* sp. LNFSFY1) (Figure 1). Thirteen mitogenomes were fully circularized, representing complete, closed-circular assemblies; the remaining three yielded linearized contigs due to unresolved terminal repeats in the control region, which is a technical limitation commonly encountered in short-read sequencing-based mitogenome reconstruction. All 16 mitogenomes contained 37 typical genes. Fourteen genes were encoded on the N-strand (8 tRNAs, 4 PCGs, and 2 rRNAs), while the remaining 23 genes were located on the J-strand. Circular genome maps for all 16 mitogenomes are presented in Figure S1.

**Figure 2:**
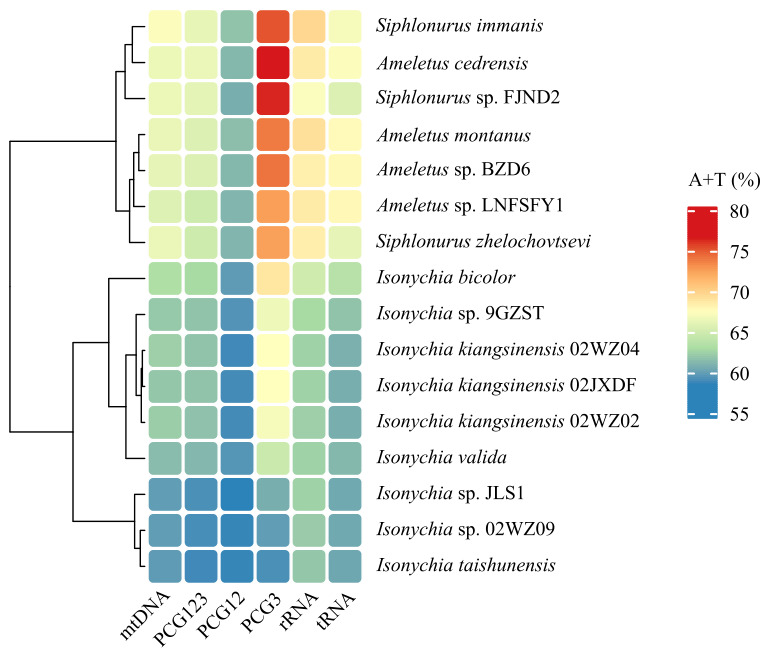
Heatmap of A+T content across different datasets of 16 Ephemeroptera mitogenomes. Hierarchical clustering of mayfly species (*y*-axis) based on A + T content. mtDNA: Mitochondrial genome; PCG123: All codon positions of the PCGs; PCG12: First and second codon positions of the PCGs; PCG3: Third codon positions of the PCGs; rRNA: Ribosomal RNA genes; tRNA: Transfer RNA genes.

**Figure 3:**
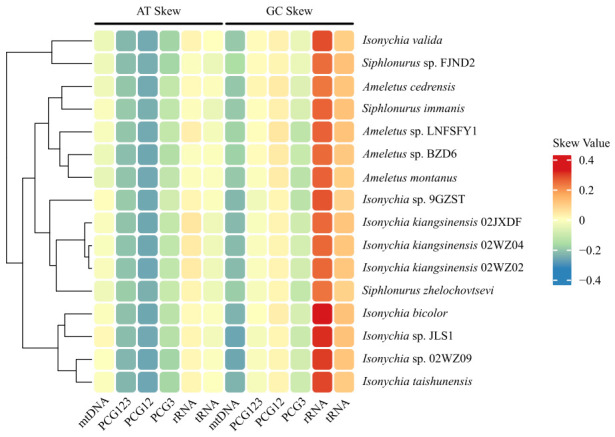
Heatmap of AT-skew and GC-skew across different datasets of 16 Ephemeroptera mitogenomes. Hierarchical clustering of mayfly species (*y*-axis) based on AT-skew and GC-skew. mtDNA: Mitochondrial genome; PCG123: All codon positions of the PCGs; PCG12: First and second codon positions of the PCGs; PCG3: Third codon positions of the PCGs; rRNA: Ribosomal RNA genes; tRNA: Transfer RNA genes.

Analysis of the 16 mitogenomes showed a typical AT bias characteristic of insects (Figure 2). The distribution of A+T content displayed significant heterogeneity across different datasets. PCG3 had the highest A+T content, ranging from 59.1% to 76.6%. PCG12 had the lowest A+T content (58.4–61.7%), indicating high conservation. Hierarchical clustering based on A+T content resolved two robust clades: one comprising all Siphlonuridae and Ameletidae specimens, characterized by elevated A+T content, particularly at PCG3 (72.5–76.6%), and a second clade consisting exclusively of Isonychiidae species, which displayed comparatively lower A+T content. Strand asymmetry analysis further demonstrated consistent negative AT-skews across all PCGs, reflecting asymmetric nucleotide composition between strands. In contrast, both rRNAs and tRNAs showed positive GC-skews (Figure 3). Notably, *ND1*, *ND4*, *ND4L*, and *ND5* genes showed negative AT-skews and positive GC-skews (Figure S2). This is because these genes are transcribed from the minority strand (N-strand).

**Figure 4:**
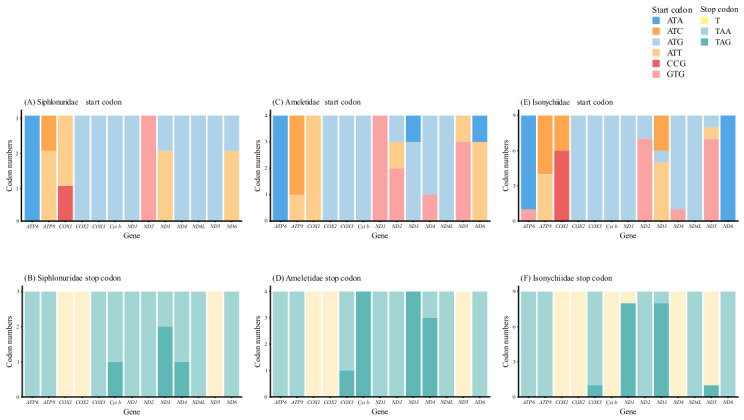
Start and stop codon usage in the PCGs of three Ephemeroptera families.

Relative synonymous codon usage (RSCU) analysis revealed lineage-specific codon preference patterns (Figure S3). UUA (L) was the most frequently used codon in both Ameletidae and Siphlonuridae, with a frequency exceeding 278 occurrences. In Isonychiidae, UUU (F) and UUA (L) exhibited the highest RSCU values. Start and stop codon usage across all 16 mitogenomes is summarized in Figure 4. Most PCGs in the three families started with typical ATN codons. The special start codon GTG was identified in *ATP6*, *ND1*, *ND2*, *ND4*, and *ND5*. The *COX1* used the special start codon CCG. Incomplete stop codons (T) were restricted to *COX1*, *COX2*, and *ND5* in Ameletidae and Siphlonuridae, and *COX1, COX2*, *Cyt b*, *ND4*, and *ND5* in Isonychiidae (Table S5).

Each newly assembled mitogenome harbored the full complement of 22 tRNA genes characteristic of metazoan mitochondrial genomes. These tRNA genes ranged in length from 59 to 71 bp. Secondary structure prediction revealed that the canonical cloverleaf conformation was conserved across the majority of tRNAs in all three families. However, structural deviations were identified in specific tRNAs: the *trnS1* (AGN) gene consistently lacked the dihydrouridine (DHU) arm across several species. Complete tRNA secondary structures for all 16 mitogenomes are presented in Figure S4.

### 3.2. Gene Arrangements

Among the 16 newly assembled mitogenomes, a conserved *trnI-trnQ-trnM-trnM* gene cluster was identified in all four Ameletidae species. In contrast, Siphlonuridae exhibited two distinct tRNA gene arrangements: a novel expanded cluster, *trnI-trnQ-trnM-trnQ-trnM-trnQ-trnM-trnQ-trnM* observed in *Siphlonurus immanis* Kluge, 1985 and *S*. sp. FJND2 and a rearranged cluster, *trnI-trnM-trnQ-trnM* found exclusively in *Siphlonurus zhelochovtsevi* Tshernova, 1952. Both the *trnI-trnQ-trnM-trnM* gene cluster in Ameletidae and the expanded *trnI-trnQ-trnM-trnQ-trnM-trnQ-trnM-trnQ-trnM* gene cluster in Siphlonuridae represent the first documented instances of these configurations within Ephemeroptera.

**Figure 5:**
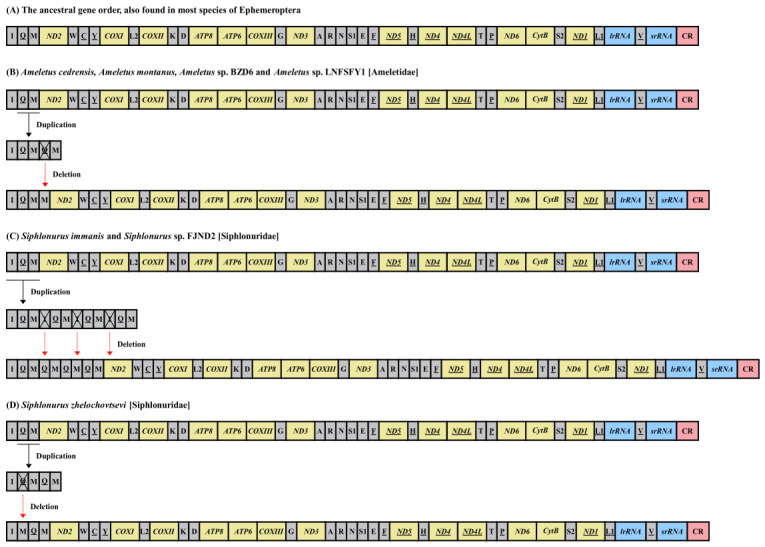
Putative gene rearrangement events in the mitogenomes of Ephemeroptera. All genes are transcribed from left to right except for those with underlines. Underlined genes have the opposite transcriptional direction. PCGs are indicated in yellow, tRNA in grey, rRNA genes in blue and control region in red. Standard abbreviations are used for PCGs, rRNAs, and control regions. The tRNAs are represented by single letters. Black dashed arrows indicate gene duplication. Red dotted arrows indicate gene loss. (**A**) The ancestral gene order, also found in most species of Ephemeroptera; (**B**) *Ameletus cedrensis*, *Ameletus montanus*, *Ameletus* sp. BZD6 and *Ameletus* sp. LNFSFY1 [Ameletidae]; (**C**) *Siphlonurus immanis* and *Siphlonurus* sp. FJND2 [Siphlonuridae]; (**D**) *Siphlonurus zhelochovtsevi* [Siphlonuridae].

Additionally, a *trnI-trnQ-trnM-trnM* gene cluster was identified in four Ameletidae mitogenomes (*Ameletus cedrensis* Sinitshenkova, 1977, *Ameletus montanus* Imanishi, 1930, *A.* sp. BZD6, and *A.* sp. LNFSFY1) (Figure 5B). This arrangement derives from the ancestral *trnI-trnQ-trnM* (Figure 5A) via tandem duplication of the *trnM* gene, resulting in an additional *trnM* inserted between the ancestral *trnM* and *ND2*.

Within the three Siphlonuridae mitogenomes, two independent rearrangement events were resolved (Figure 5C,D). The first type occurred in *Siphlonurus immanis* and *S*. sp. FJND2: arose from iterative tandem duplications of the *trnQ-trnM* unit downstream of *trnI-trnQ-trnM-ND2*, yielding the nine-gene cluster *trnI-trnQ-trnM-trnQ-trnM-trnQ-trnM-trnQ-trnM* (Figure 5C). The second type was found in the *S*. *zhelochovtsevi:* resulted from a single *trnM* insertion between *trnI* and *trnQ*, producing the *trnI-trnM-trnQ-trnM* configuration (Figure 5D).

### 3.3. Phylogeny Analyses

**Figure 6:**
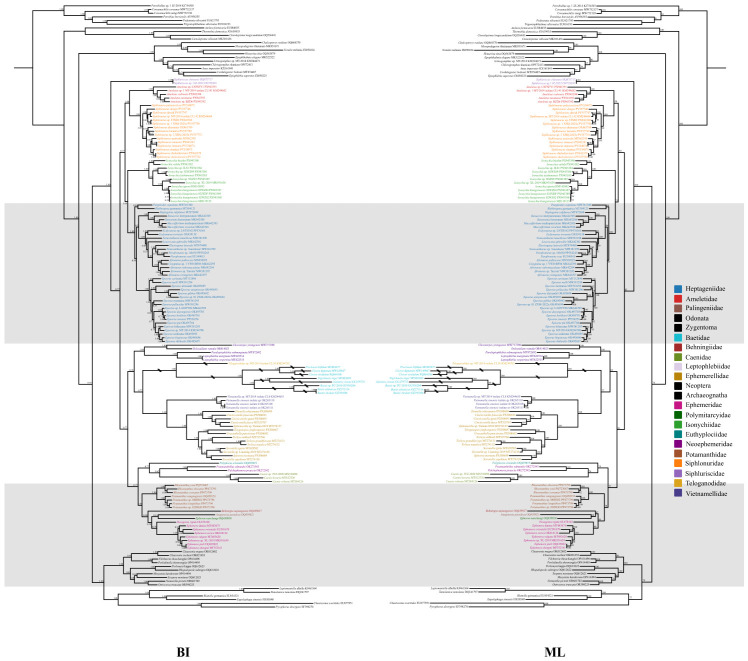
BI tree (**left**) and ML tree (**right**) of Archaeognatha, Zygentoma, Odonata, Ephemeroptera, and Neoptera constructed based on PCG12. Six species of Archaeognatha were used as outgroups. The numbers on the nodes represent the bootstrap values of the ML tree and the posterior probabilities of the BI tree. The shaded areas indicate regions with topological inconsistencies.

**Figure 7:**
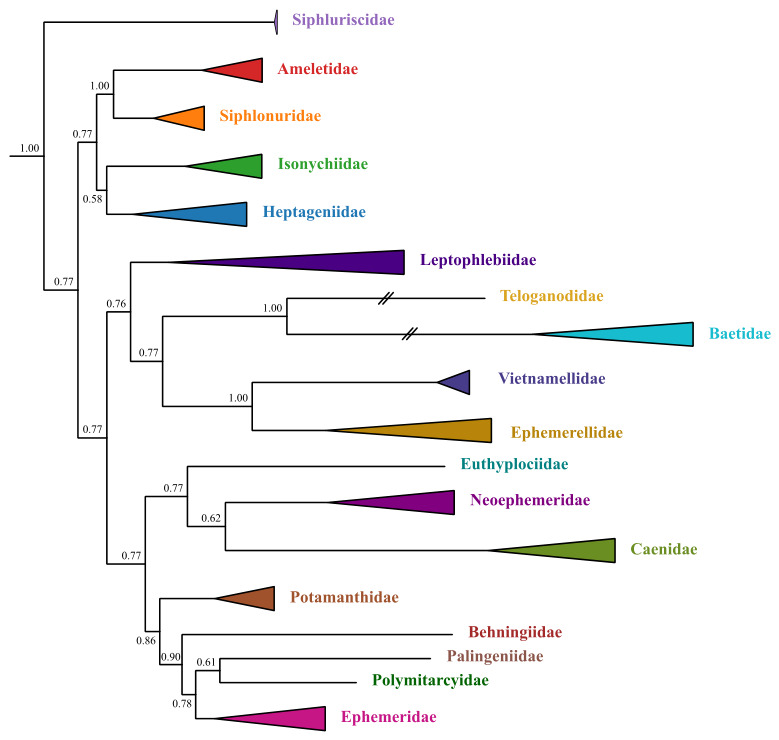
Summary hypothesis of the phylogenetic relationships of major Ephemeroptera families inferred from BI tree.

Phylogenetic inference based on the PCG12 dataset yielded two topologies through Bayesian inference (BI) and maximum likelihood (ML) analyses (Figure 6). These two trees exhibit certain topological incongruences, primarily concerning the relationships among Behningiidae, Euthyplociidae, Palingeniidae, and Ephemeridae. Minor topological discrepancies also occur within the Plecoptera, as well as within Heptageniidae and Potamanthidae of Ephemeroptera, though these did not affect higher-level relationships. Since the BI tree was more robust and aligned better with morphological taxonomy, the summary hypothesis of the phylogenetic relationships of major Ephemeroptera families was based on this BI topology (Figure 7).

Siphluriscidae is resolved as the sister lineage to all other Ephemeroptera, occupying the most basal position in the order. Ameletidae and Siphlonuridae tend to form a clade with strong statistical support, whereas Isonychiidae and Heptageniidae together constitute another distinct lineage, with these two clades standing as sister groups to each other. The assemblage composed of these four families constitutes a cluster, which appears to be a sister group to the cluster containing the remaining thirteen families, excluding Siphluriscidae.

The remaining thirteen families together constitute a large clade containing two major subclades. Within the first subclade, the topology is expressed as (Leptophlebiidae + ((Teloganodidae + Baetidae) + (Vietnamellidae + Ephemerellidae))). The topology of the second subclade is expressed as ((Euthyplociidae + (Neoephemeridae + Caenidae)) + (Potamanthidae + (Behningiidae + ((Palingeniidae + Polymitarcyidae) + Ephemeridae)))). Long-branch attraction (LBA) was detected between Baetidae and Teloganodidae. Site-heterogeneity tests revealed high levels of heterogeneity in these two families (Figure S5). The *trnI-trnQ-trnM-trnM* gene cluster was identified in all four Ameletidae mitogenomes. In contrast, gene order variation within Siphlonuridae was incongruent with phylogenetic signal. *Siphlonurus immanis* and *S.* sp. FJND2 share the expanded *trnI-trnQ-trnM-trnQ-trnM-trnQ-trnM-trnQ-trnM* gene arrangement. Yet they do not form a monophyletic group. Instead, *S. immanis* clusters with *S. zhelochovtsevi*, which harbors the distinct *trnI-trnM-trnQ-trnM* configuration This topological discordance suggests that the gene cluster arose independently in *S. immanis* and *S*. sp. FJND2 or was subject to subsequent homoplasy rather than reflecting shared ancestry.

### 3.4. Estimation of Divergence Time

**Figure 8:**
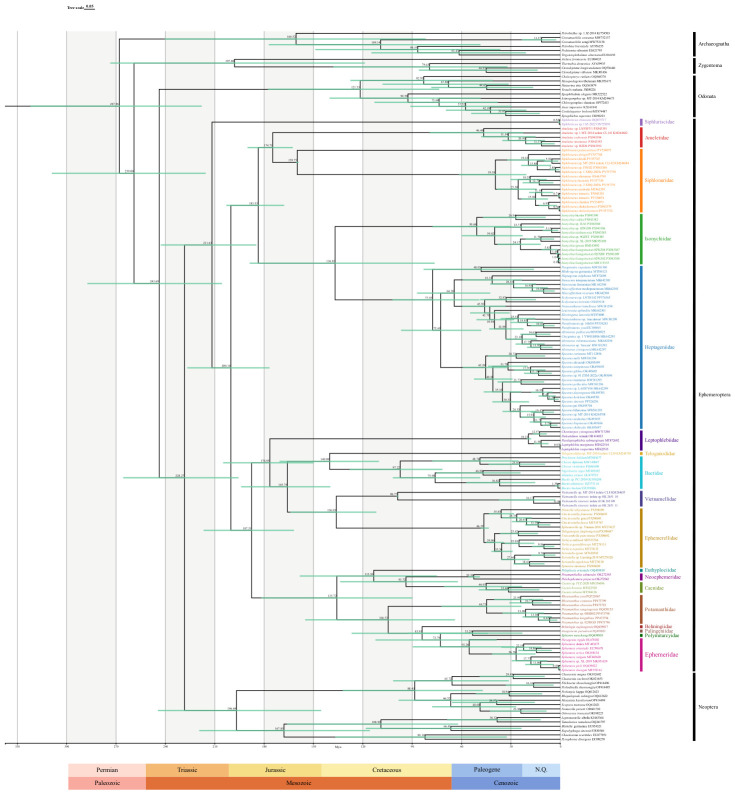
Divergence times of Archaeognatha, Zygentoma, Odonata, Ephemeroptera and Neoptera based on the phylogenetic tree and five fossil calibration points. The numbers above the nodes indicate the median ages.

Divergence times (Figure 8) were estimated based on the BI topology. Crown-group Ephemeroptera originated during the Late Triassic (211.61 Mya, 95% HPD: 184.65–242.74 Mya). The major radiation of extant families occurred predominantly during the Cretaceous, spanning approximately 140.09 to 71.79 Mya. 

Siphluriscidae was resolved as the sister lineage to all other Ephemeroptera, diverging in the Late Triassic (211.61 Mya, 95% HPD: 184.65–242.74 Mya). The split between Ameletidae and Siphlonuridae was dated to the Early Jurassic (174.71 Mya, 95% HPD: 162.45–190.03 Mya), while Isonychiidae and Heptageniidae diverged later, during the Early Cretaceous (136.81 Mya, 95% HPD: 76.27–188.72 Mya). Among the remaining 13 families, two primary diversification pulses were identified: an Early Jurassic origin (176.45 Mya, 95% HPD: 149.91–204.87 Mya) for a clade comprising five families, and an Early Cretaceous origin (135.72 Mya, 95% HPD: 89.71–181.62 Mya) for the remaining eight families. All node ages, including posterior distributions and support metrics, are reported in Table S6.

## 4. Discussion

### 4.1. Characteristics of Ephemeroptera Mitogenomes

This study reports 16 newly assembled mitochondrial genome sequences from 14 species across three families of Ephemeroptera. Among these 14 species, 11 have their mitochondrial genomes deposited in NCBI for the first time, including five species belonging to Siphlonuridae and Ameletidae. These newly generated data significantly broaden the phylogenetic sampling within Siphlonuroidea and substantially augment the available mitochondrial genomic resources for the order Ephemeroptera. The gene composition and genome lengths of the new sequences fell within the typical range for Ephemeroptera [13,20,21,33,34,35,37,54,63,65,67,68,112,113,114]. Mean mitogenome length was 16,122 bp in Ameletidae and 16,089 bp in Siphlonuridae, both exceeding the Isonychiidae average of 15,576 bp. This size divergence is attributable to lineage-specific expansions in the control region and intergenic spacers (IGSs), as well as structural rearrangements including tRNA duplications and positional shifts, documented across multiple insect orders [115,116,117].

Hierarchical clustering based on A+T content resolved two major clades: one uniting all Siphlonuridae and Ameletidae specimens, and a second comprising exclusively Isonychiidae species. This partition reflects pronounced compositional divergence: Siphlonuridae and Ameletidae share elevated and highly congruent A+T content across genomic partitions (particularly at PCG3), whereas Isonychiidae exhibits significantly lower and more constrained values. As proposed by Cameron, such directional shifts in nucleotide composition often constitute molecular synapomorphies shared derived traits supporting monophyly [22]. The reduced A+T content in Isonychiidae thus represents a lineage-specific evolutionary shift, reinforcing its phylogenetic distinction from the (Siphlonuridae + Ameletidae) clade.

Strand asymmetry analysis revealed consistent negative AT-skew and negative GC-skew across the majority of PCGs, reflecting asymmetric mutational pressure during mitochondrial DNA replication [118]. Structural modeling further confirmed that *trnS1* (AGN) lacks the DHU arm in multiple species, a feature recurrently observed across diverse insect lineages [29,119,120] and functionally compensated via post-transcriptional modifications. This conservation of translational functionality despite structural simplification underscores the robust coevolutionary capacity of the mitochondrial translation apparatus [121].

### 4.2. Gene Arrangement Patterns and Mechanisms

The *trnI-trnQ-trnM* gene cluster is a well-documented hotspot for mitochondrial gene rearrangement across Insecta, with recurrent structural variation reported in multiple orders [122,123] and frequent occurrence in Ephemeroptera [21,35,36,37]. In this study, three types of gene rearrangements were identified within this locus across the sampled ephemeropteran mitogenomes (Figure 5B–D), all representing lineage-specific modifications of the ancestral *trnI-trnQ-trnM-ND2* arrangement (Figure 5A). The tandem duplication-random loss (TDRL) model [124] is commonly used to explain rearrangements and duplications of PCGs, CR, and tRNAs, and is also applied to similar phenomena in other insect groups [38,117,122,125,126,127,128]. The TDRL model provides a plausible explanation for the three gene rearrangement mechanisms identified in this study.

An identical *trnI-trnQ-trnM-trnM* gene cluster was identified in four Ameletidae species (*Ameletus cedrensis, A. montanus, A.* sp. BZD6, and *A.* sp. LNFSFY14). The rearrangement likely stems from the ancestral *trnI-trnQ-trnM* template (Figure 5A). A tandem duplication of *trnQ-trnM* produced the *trnI-trnQ-trnM-trnQ-trnM* intermediate. The subsequent random loss of the second *trnQ* yielded the *trnI-trnQ-trnM-trnM* gene cluster (Figure 5B). The occurrence of this gene cluster in four newly sequenced species suggests that this rearrangement pattern is a potential synapomorphy for Ameletidae. Further sampling of Ameletidae is necessary to verify this hypothesis in future studies.

Two distinct tRNA gene order configurations were identified within Siphlonuridae. The *trnI-trnQ-trnM-trnQ-trnM-trnQ-trnM-trnQ-trnM* gene cluster identified in *Siphlonurus immanis* and *S.* sp. FJND2 represents the first record for Siphlonuridae. This arrangement derives from the ancestral *trnI-trnQ-trnM* gene cluster through three tandem duplications. This process produced a *trnI-trnQ-trnM-trnI-trnQ-trnM-trnI-trnQ-trnM-trnI-trnQ-trnM* gene cluster. The subsequent random loss of the three newly generated *trnI* yielded the final gene order (Figure 5C). Notably, this arrangement differs from that reported for a previously sequenced *S. immanis* specimen (GenBank accession PV534074) [60], which harbors the *trnI-trnM-trnQ-trnM* gene cluster. Despite 99.4% sequence identity across coding regions, the discordant gene orders coupled with absence of morphological or ecological divergence preclude cryptic speciation as an explanation. Instead, this represents intraspecific mitochondrial gene order polymorphism. Such occurrences have been documented in previous studies. Sweet et al. reported heterogeneity in mitochondrial gene arrangement among eleven individuals of *Columbicola passerinae* [129]. Additionally, in a study of *Icerya aegyptiaca*, Ye et al. demonstrated that the presence of long tandem repeats leads to divergence in both gene copy number and order among three haplotypes [130]. The differences observed in the *trnI-trnQ-trnM* rearrangement hotspot of *S. immanis* likely represent TDRL-mediated gene rearrangement polymorphism at the intraspecific level within Ephemeroptera.

An alternative rearrangement pattern, *trnI-trnM-trnQ-trnM*, occurs in the *Siphlonurus zhelochovtsevi* mitogenome. arrangement is phylogenetically widespread, having been reported in multiple Siphlonuridae species [60] and Heptageniidae [21]. It likely originates from the ancestral *trnI-trnQ-trnM* gene cluster via a single tandem duplication. The subsequent random loss of the newly formed *trnQ* results in the final *trnI-trnM-trnQ-trnM* gene cluster (Figure 5D). Collectively, these findings reveal substantial gene order diversity within Siphlonuridae, not only across species but also within a single nominal species.

### 4.3. Phylogenetic Analyses

This study presents a relatively comprehensive mitogenomic phylogenetic framework of Ephemeroptera, encompassing 18 families and providing new insights into the relationships among families within this order (Figure 7). The research aims to clarify the taxonomic placement of families within Siphlonuroidea and reconstructed the phylogenetic status of Siphlonuroidea within Ephemeroptera.

Siphluriscidae was recovered as the sister lineage to all other Ephemeroptera, occupying the most basal position in the inferred phylogeny. This family occupies the basal position in the Ephemeroptera phylogeny. This result is congruent with numerous prior molecular and morphological studies [8,12,13] and justifies its frequent use as an outgroup in ephemeropteran phylogenetics [20,21,34,35]. Collectively, these findings good evidence Siphluriscidae as representing the earliest diverging extant lineage within crown-group Ephemeroptera and as a lineage that falls outside the Siphlonuroidea clade.

Among the remaining 17 families, a clade comprising Ameletidae, Siphlonuridae, Isonychiidae, and Heptageniidae was resolved with good support as sister to the remaining 13 families. This topology aligns with the evolutionary hypotheses proposed by Ogden et al. [8] and Yu et al. [13]. Within this clade, reciprocal monophyly was recovered for two subclades: (Ameletidae + Siphlonuridae) and (Isonychiidae + Heptageniidae), a relationship corroborated by independent evidence McCafferty [131], Kluge [132], Ogden et al. [8], and Yu et al. [13]. In contrast, this arrangement conflicts with alternative topologies reported by Ogden et al. [12], Guo et al. [21,34], and Tong et al. [20], who recovered Isonychiidae as the second-branching lineage after Siphluriscidae without affiliation to Heptageniidae. The good support for the (Isonychiidae + Heptageniidae) clade in our mitogenomic analysis suggests that previous discordant placements might be related to methodological limitations instead of entirely reflecting true biological phenomena. Notably, the close phylogenetic affinity between Isonychiidae and Heptageniidae further calls into question the traditional view that includes Isonychiidae within the superfamily Siphlonuroidea. Based on these findings, we tentatively speculate that the traditionally defined Siphlonuroidea [5,6], which encompasses Isonychiidae, might not represent a monophyletic group, and that Ameletidae and Siphlonuridae may instead constitute the core lineage of this superfamily. This inference is consistent with the taxonomic scheme proposed by Kluge et al. [1], who defined Siphlonuroidea as a group comprising 10 families that excludes Isonychiidae. Nevertheless, due to constraints in currently available public data, even after integrating our newly sequenced mitogenomes with public sequences from the NCBI database, our taxonomic sampling within Siphlonuroidea remains restricted to Ameletidae and Siphlonuridae. Consequently, the conclusion proposed based on our present data should be interpreted as a preliminary hypothesis. Furthermore, phylogenomic inference based solely on mitochondrial genomes may possess inherent limitations and might not fully capture complex evolutionary histories. Therefore, a more extensive taxon sampling combined with the integration of multilocus markers or nuclear genomic data is warranted in future studies to further elucidate the phylogenetic relationships within Siphlonuroidea.

Furthermore, the topology (Archaeognatha + (Zygentoma + (Odonata + (Ephemeroptera + Neoptera)))) emerged at the ordinal level. This arrangement appears to support the Chiastomyaria hypothesis [13,14,133] rather than the widely accepted Paleoptera hypothesis [18,134,135]. This phenomenon might stem from the inherent limitations of mitochondrial genomes. Mitochondrial sequences frequently demonstrate accelerated substitution rates and base compositional biases. These molecular characteristics can easily induce long branch attraction artifacts during deep evolutionary reconstructions [133]. Therefore, comprehensive nuclear genomic data remains essential in the future to deeply investigate the phylogenetic relationships among insect orders.

Mitochondrial gene rearrangements are frequently proposed as phylogenetically informative characters due to their presumed rarity and irreversibility [38,39]. However, this study reveals a case of phylogenetic incongruence between gene order and sequence-based topology highlighting the risk of homoplasy in tRNA cluster evolution. *Siphlonurus immanis* and *S.* sp. FJND2 share an identical *trnI-trnQ-trnM-trnQ-trnM-trnQ-trnM-trnQ-trnM* gene cluster, yet they do not form a monophyletic clade. *S. immanis* shows a closer phylogenetic affinity to *S. zhelochovtsevi*, which has a *trnI-trnM-trnQ-trnM* gene cluster. This topological discordance is best explained by convergent homoplasy rather than shared ancestry. Such convergence is empirically supported by Dowton’s analysis of Hymenoptera, which documented 67 independent rearrangement events, of which only two were validated as genuine synapomorphies despite recurrence in multiple lineages [40]. Similarly, the *trnI-trnM-trnQ-trnM* rearrangement exists in both Siphlonuridae [60] and Heptageniidae [21], strongly suggesting independent derivation, a pattern analogous to the well-documented convergent evolution of the *trnD-trnK* rearrangement, which arose separately in Acridomorpha [136] and five distinct hymenopteran families [40,137,138,139,140]. While lineage-specific rearrangements remain the predominant pattern, our findings underscore that convergence in tRNA gene order, particularly within hotspots such as the *trnI-trnQ-trnM* locus, is a non-negligible source of phylogenetic noise and must be rigorously evaluated against sequence-based evidence. This study expands the mitochondrial genome datasets for Ameletidae, Siphlonuridae, and Isonychiidae. These new data facilitate an understanding of the evolutionary history and phylogenetic relationships within Siphlonuroidea.

This study estimates the origin of crown-group Ephemeroptera in the Late Triassic, consistent with fossil-calibrated molecular dating analyses by García-Girón et al. [19], which place the earliest divergences within the order during the Triassic. At the family level, Siphlonuridae diverges in the Early Jurassic, a timeframe corroborated by fossil evidence from *Cheirolgisca ningchengensis* Lin & Huang, 2008 and *Olgisca angusticubitis* Lin & Huang, 2008, both dated to the Late Jurassic (159.00–160.60 Mya) [108]. The divergence of Isonychiidae is estimated in the Early Cretaceous, aligning with García-Girón’s estimate [19]. Collectively, our analyses indicate that the major radiation of extant ephemeropteran families occurred predominantly during the Cretaceous, supporting the hypothesis that this period constituted a critical phase of diversification for the order [16,19,21].

## 5. Conclusions

This study explores the phylogenetic relationships and evolutionary history within Siphlonuroidea through integrated phylogenomic, gene order, and divergence time analyses of 16 newly assembled mitogenomes from 14 species. Two distinct tRNA gene configurations were identified within Siphlonuridae, whereas all four Ameletidae species share the same gene rearrangement pattern. The phylogenetic analysis based on the PCG12 dataset provides good support for a ((Ameletidae + Siphlonuridae) + (Isonychiidae + Heptageniidae)) topology. Divergence time estimation calibrated by five fossil constraints indicates that Ameletidae and Siphlonuridae diverged during the Early Jurassic, whereas Isonychiidae originated in the Early Cretaceous. These congruent lines of evidence demonstrate that Ameletidae and Siphlonuridae form a well-supported clade consistent with superfamily status, whereas Isonychiidae is phylogenetically allied with Heptageniidae.

## Figures and Tables

**Table 1:** Detailed information on the 16 sequences from 14 species used in this study.

Specimen No.	Family	Species	Length	Sampling Localities	Accession No.
HLJFY2	Siphlonuridae	*Siphlonurus immanis*	16,138	Haerbin China	PX943381
JLFY14	Siphlonuridae	*Siphlonurus zhelochovtsevi*	16,006	Jilin, China	PX943379
FJND2	Siphlonuridae	*Siphlonurus* sp. FJND2	15,939	Ningde, China	PX943380
JLFY13	Ameletidae	*Ameletus cedrensis*	16,454	Jilin, China	PX943394
NLTYT3	Ameletidae	*Ameletus montanus*	16,671	Yili, China	PX943393
LNFSFY1	Ameletidae	*Ameletus* sp. LNFSFY1	16,687	Fushun, China	PX943391
BZD6	Ameletidae	*Ameletus* sp. BZD6	15,280	Wensu, China	PX943392
DXY3	Isonychiidae	*Isonychia taishunensis*	16,484	Wenzhou, China	PX943383
JLH11	Isonychiidae	*Isonychia valida*	15,566	Jilin, China	PX943382
03FY38	Isonychiidae	*Isonychia bicolor*	15,084	Ottawa, Canada	PX943390
02JXDF	Isonychiidae	*Isonychia kiangsinensis* 02JXDF	15,107	Jinhua, China	PX943389
02WZ02	Isonychiidae	*Isonychia kiangsinensis* 02WZ02	15,597	Wenzhou, China	PX943388
02WZ04	Isonychiidae	*Isonychia kiangsinensis* 02WZ04	15,592	Wenzhou, China	PX943387
JLS1	Isonychiidae	*Isonychia* sp. JLS1	15,960	Jiujiang, China	PX943384
02WZ09	Isonychiidae	*Isonychia* sp. 02WZ09	15,342	Wenzhou, China	PX943386
9GZST	Isonychiidae	*Isonychia* sp. 9GZST	15,453	Tongren, China	PX943385

**Table 2:** Fossil calibration points used for divergence time estimation in this study.

Dated Crown Group	Calibration Fossil	MCMCTree Bounds (Mya)	Phylogenetic Justification
Crown Vietnamellidae	*Burmella* sp.	98.17-99.41	[107]
Crown Siphlonuridae	*Cheirolgisca ningchengensis*	159.00-160.60	[108]
Crown Ephemerellidae	*Eurylophella viscata*	41.30-47.80	[109]
Crown Leptophlebiidae	*Borinquena schawallfussi*	15.00-20.00	[110]
Crown Neoephemeridae	*Neoephemera* sp.	48.60-55.80	Fossilworks

## Data Availability

Data to support this study are available from the National Center for Biotechnology Information (https://www.ncbi.nlm.nih.gov) (accessed on 11 May 2026). GenBank numbers are PX943379-PX943394.

## Supplementary Materials

The following supporting information can be downloaded at: https://www.mdpi.com/article/10.3390/insects17070718/s1, Figure S1: Structural characteristics of the 16 newly sequenced mitogenomes. Figure S2: Box-and-whisker plots for nucleotide composition of each gene and mitogenome. (A) Siphlonuridae AT-skew; (B) Ameletidae AT-skew; (C) Isonychiidae AT-skew; (D) Siphlonuridae GC-skew; (E) Ameletidae GC-skew; (F) Isonychiidae GC-skew. Figure S3. The RSCU of the 16 newly sequenced mitogenomes. Figure S4: The secondary structure of tRNAs in the 16 newly sequenced mitogenomes. Figure S5. Heterogeneity test of the concatenated dataset consisting of the first and second codon positions (PCG12) of protein-coding genes from 164 insect mitochondrial genomes. Table S1: Species used in this study for phylogenetic reconstruction and their NCBI GenBank accession numbers. Table S2: The results of codon saturation analysis for the first, second, and third codon positions of protein coding genes in 164 mitochondrial genomes. Table S3: Best partitioning schemes and best evolutionary models selected by PartitionFinder v.2.1.1. Table S4: Best partitioning schemes and best evolutionary models selected by ModelFinder. Table S5: Location of features of the 16 newly sequenced mitogenomes. Table S6: The divergence-time of each family.
