# Supplementary material for: Comparative Mitogenomics Reveals Gene Rearrangement and Phylogenetic Relationships in Siphlonuroidea (Insecta: Ephemeroptera)
Source: Insects. 2026 Jul 11;17(7):718. doi: 10.3390/insects17070718 (PMC13410250; doi:10.3390/insects17070718)
Supplement: Supplementary file 1 [file insects-17-00718-s001.zip › Figure S1.pdf]

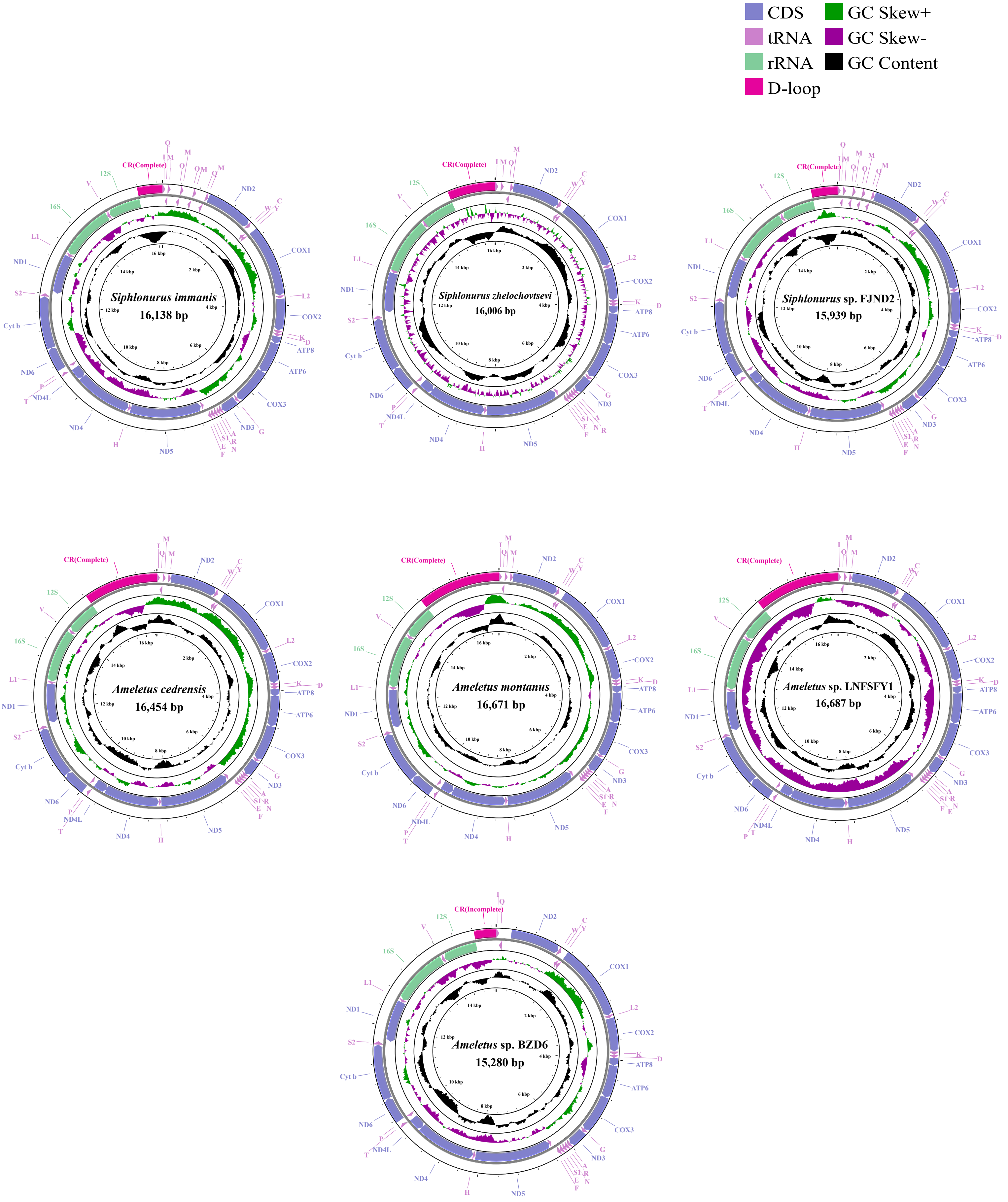

Figure S1. Structural characteristics of the 16 newly sequenced mitogenomes.

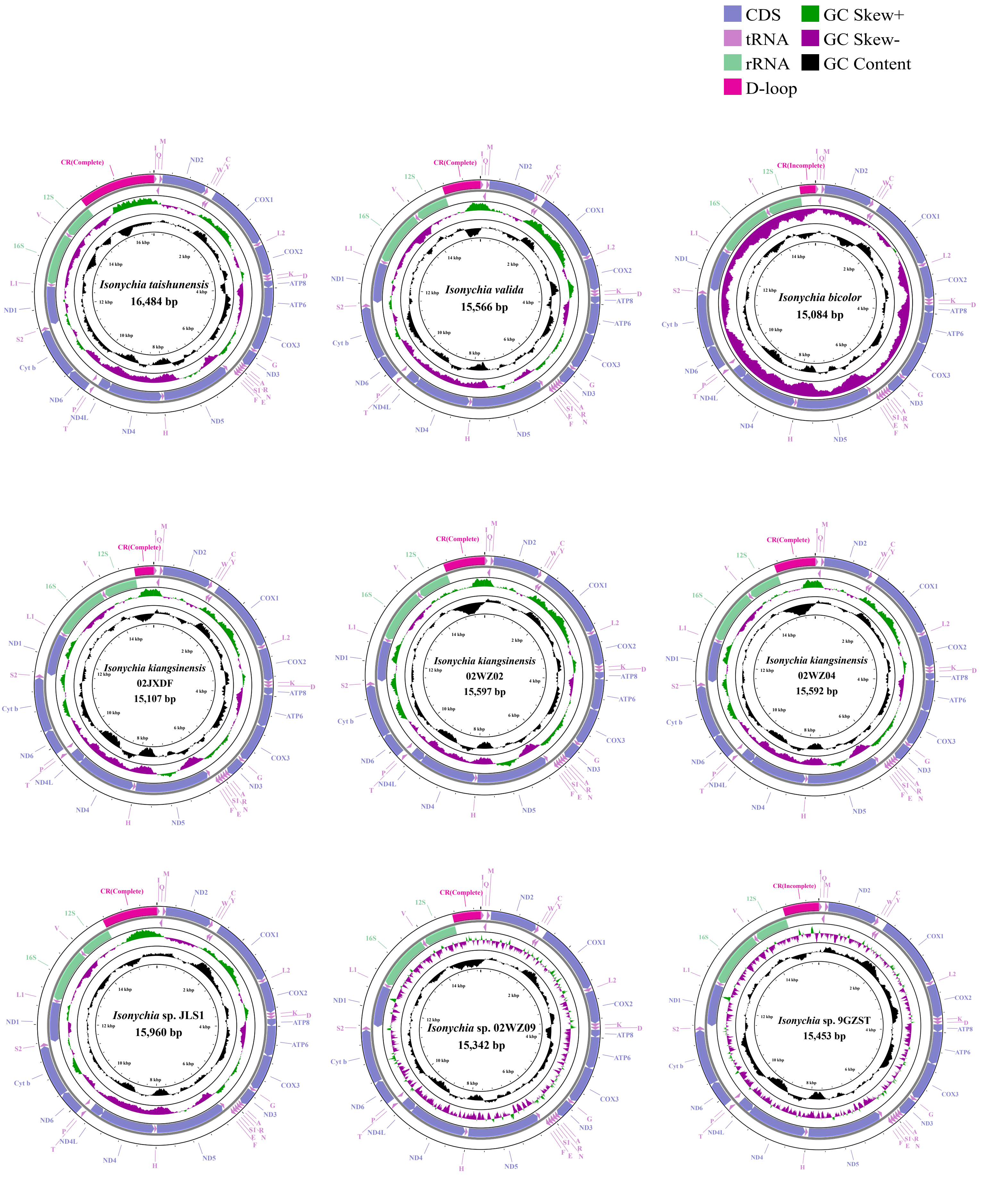

Figure S1. Structural characteristics of the 16 newly sequenced mitogenomes.
