# Supplementary material for: Comparative Mitogenomics Reveals Gene Rearrangement and Phylogenetic Relationships in Siphlonuroidea (Insecta: Ephemeroptera)
Source: Insects. 2026 Jul 11;17(7):718. doi: 10.3390/insects17070718 (PMC13410250; doi:10.3390/insects17070718)
Supplement: Supplementary file 1 [file insects-17-00718-s001.zip › Figure S2.pdf]

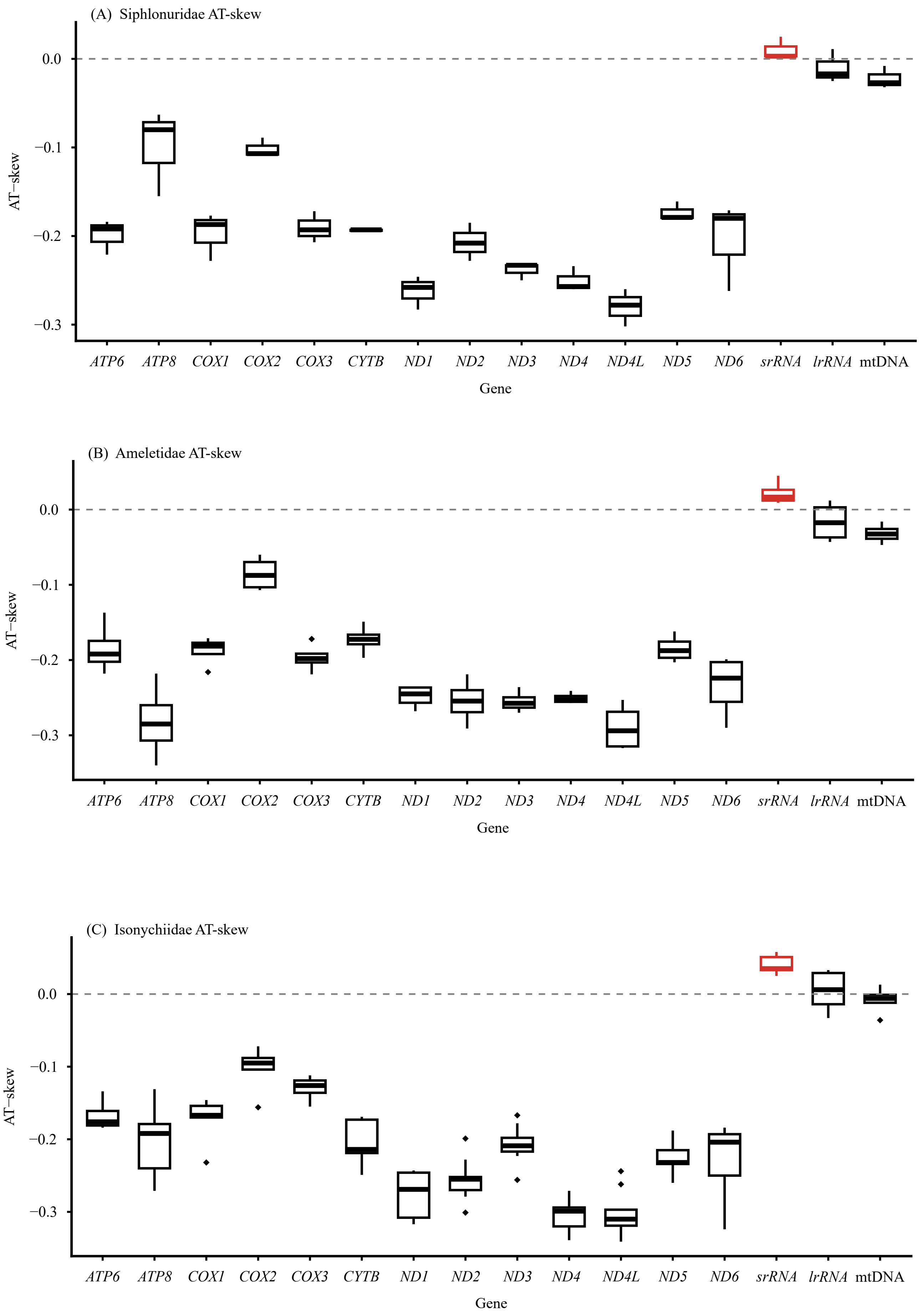

Figure S2. Box-and-whisker plots for nucleotide composition of each gene and mitogenome.

(A) Siphlonuridae AT-skew; (B) Ameletidae AT-skew; (C) Isonychiidae AT-skew;

(D) Siphlonuridae GC-skew; (E) Ameletidae GC-skew; (F) Isonychiidae GC-skew.

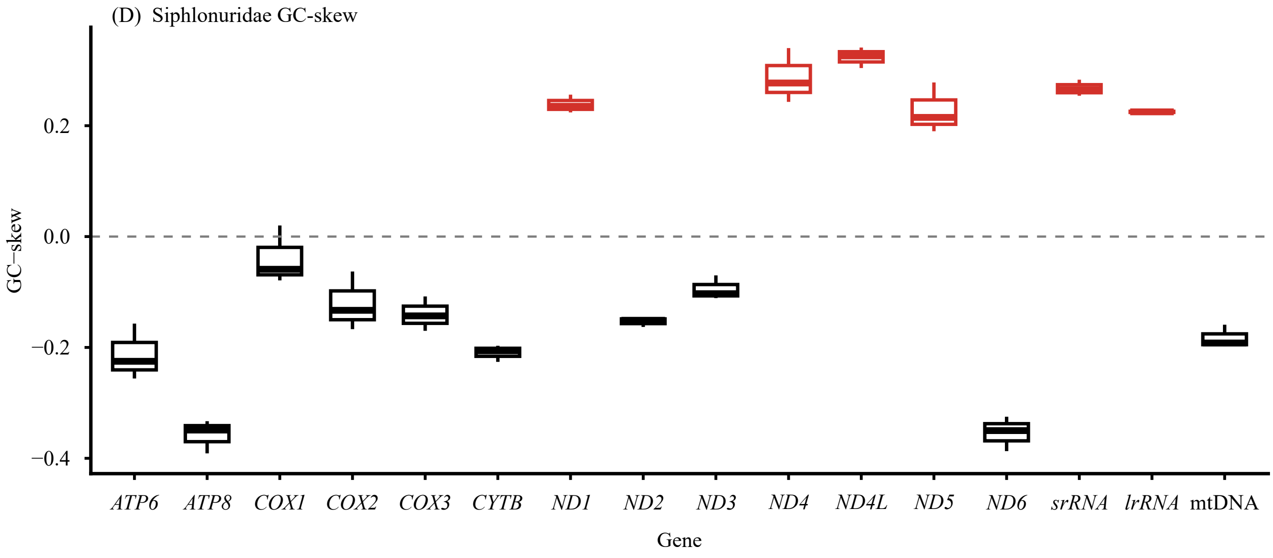

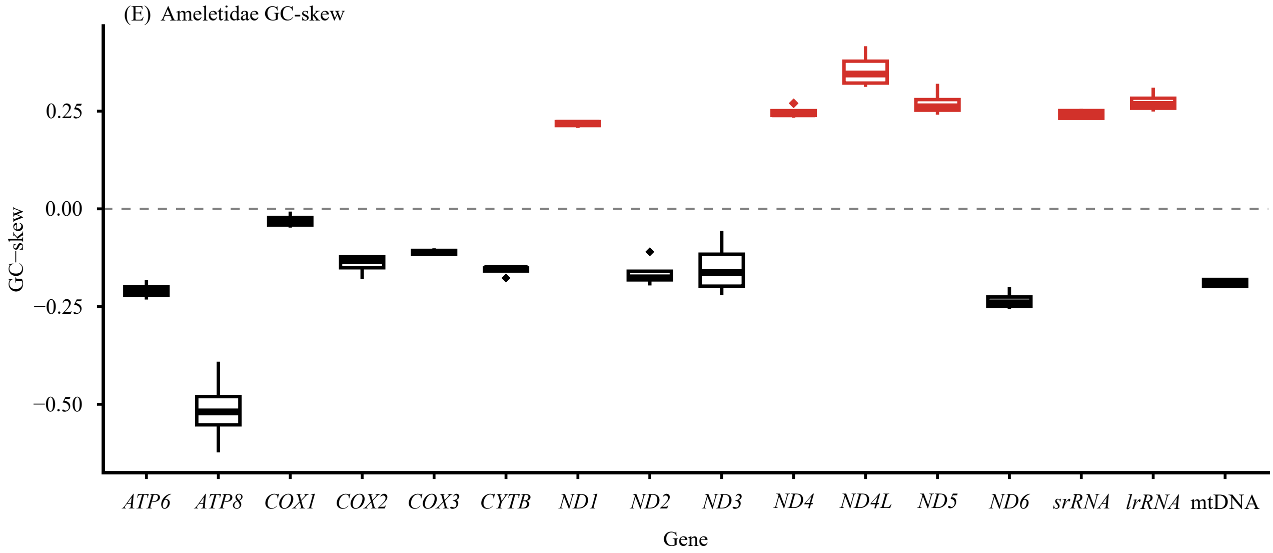

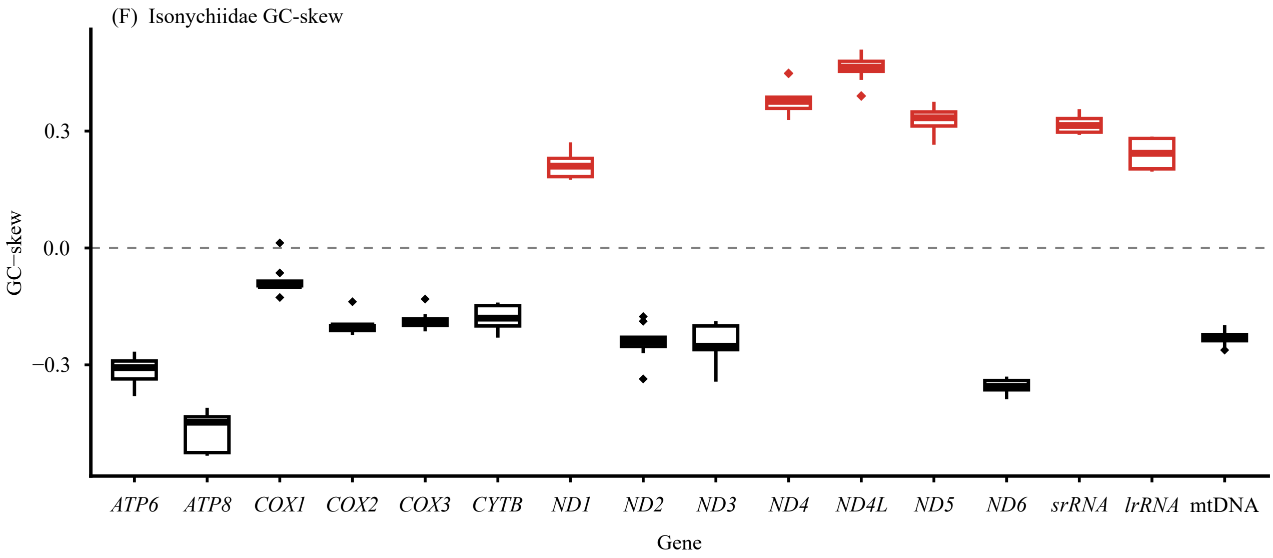

Figure S2. Box-and-whisker plots for nucleotide composition of each gene and mitogenome.

(A) Siphlonuridae AT-skew; (B) Ameletidae AT-skew; (C) Isonychiidae AT-skew;

(D) Siphlonuridae GC-skew; (E) Ameletidae GC-skew; (F) Isonychiidae GC-skew.
