# Supplementary material for: Comparative Mitogenomics Reveals Gene Rearrangement and Phylogenetic Relationships in Siphlonuroidea (Insecta: Ephemeroptera)
Source: Insects. 2026 Jul 11;17(7):718. doi: 10.3390/insects17070718 (PMC13410250; doi:10.3390/insects17070718)
Supplement: Supplementary file 1 [file insects-17-00718-s001.zip › Figure S4.pdf]

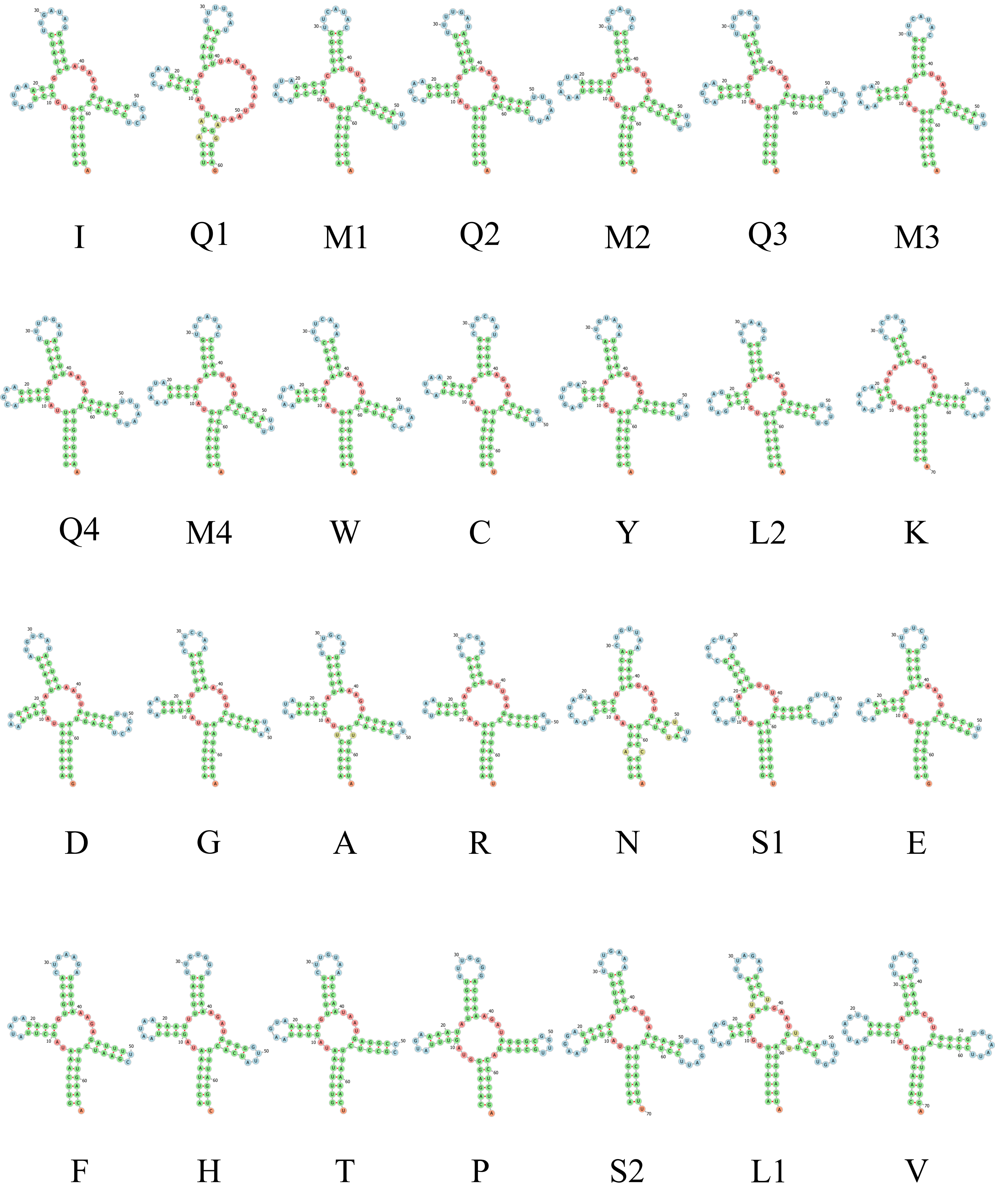

(A) *Siphlonurus immanis*

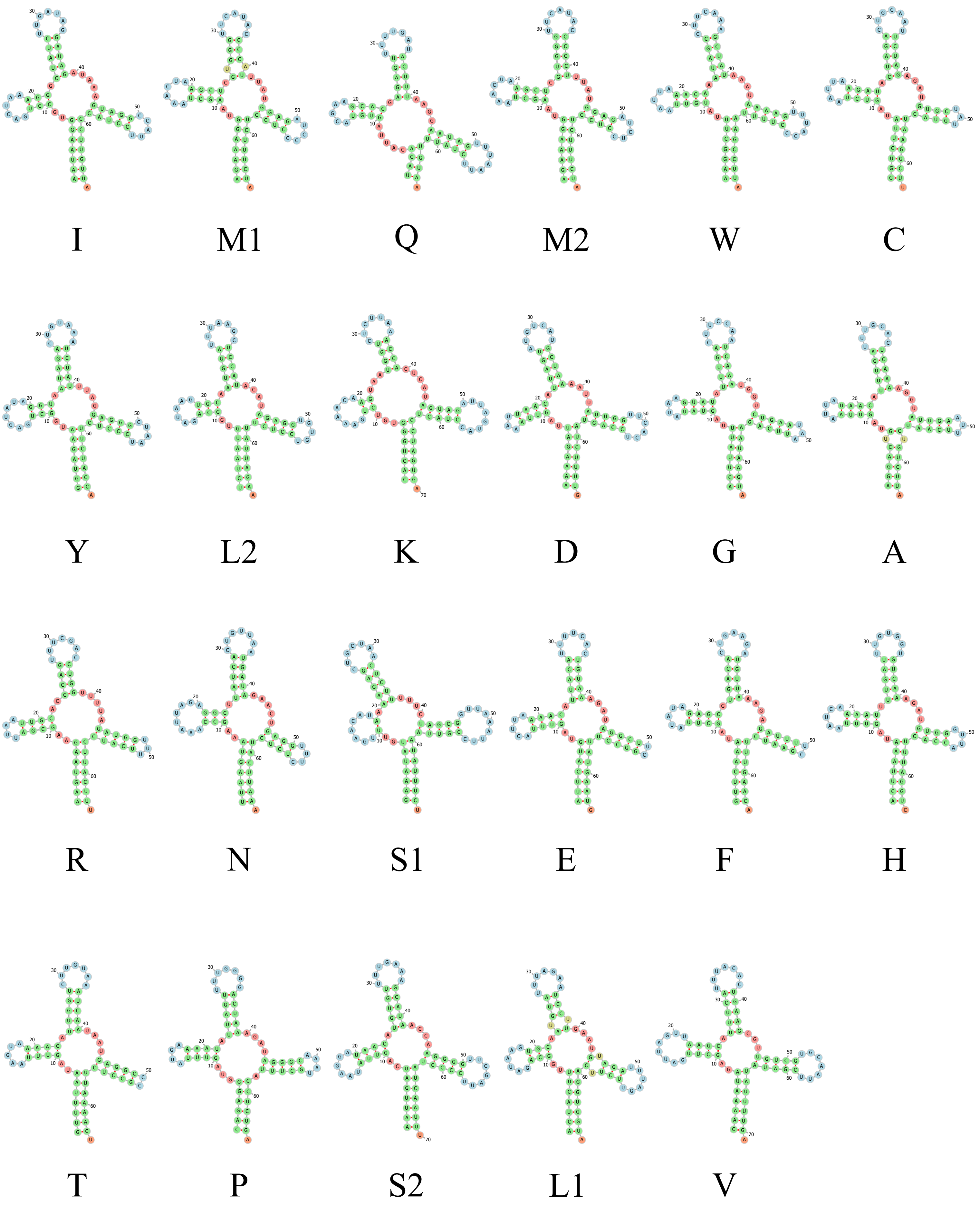

(B) *Siphlonurus zhelochovtsevi*

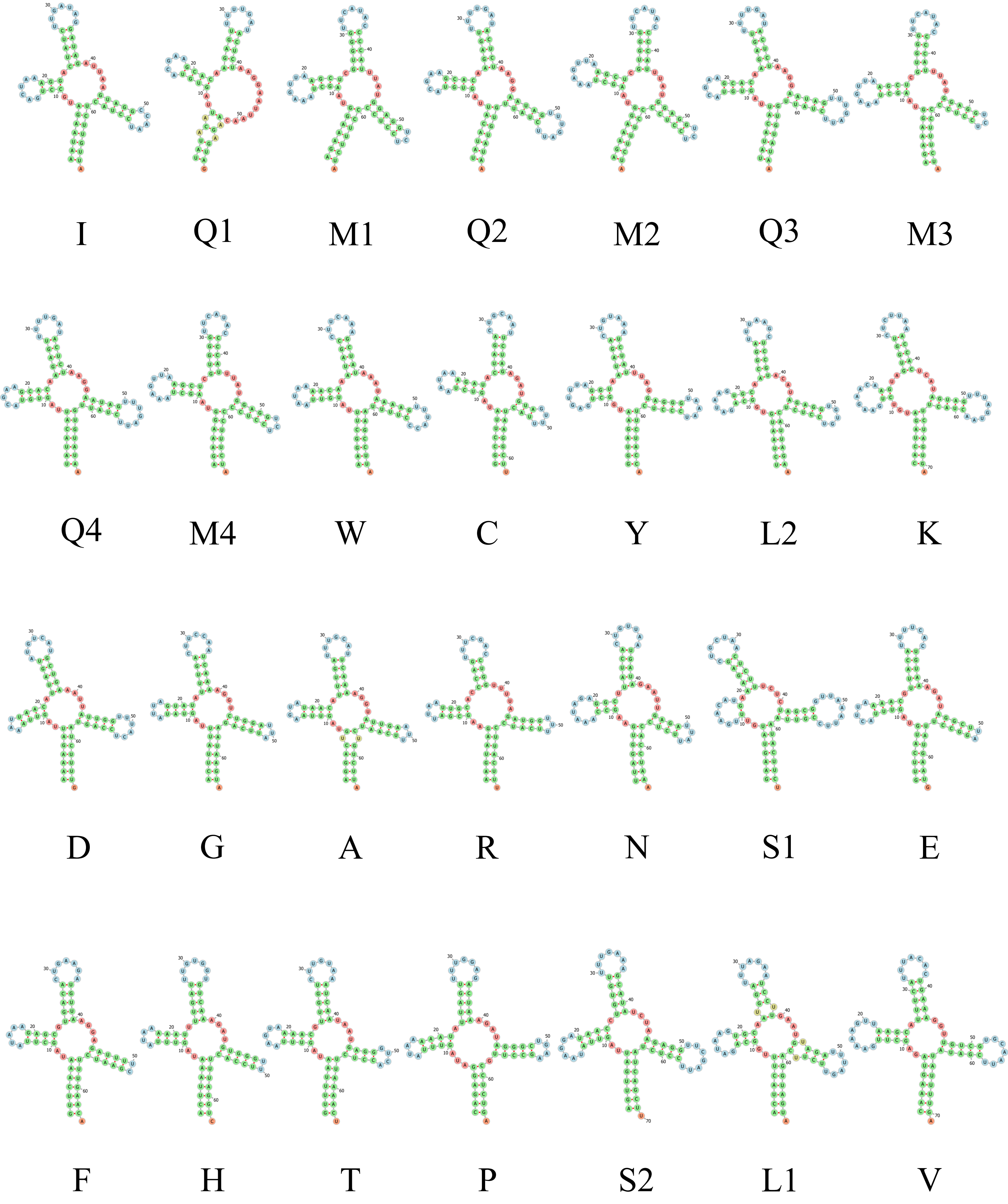

(C) *Siphlonurus* sp. FJND2

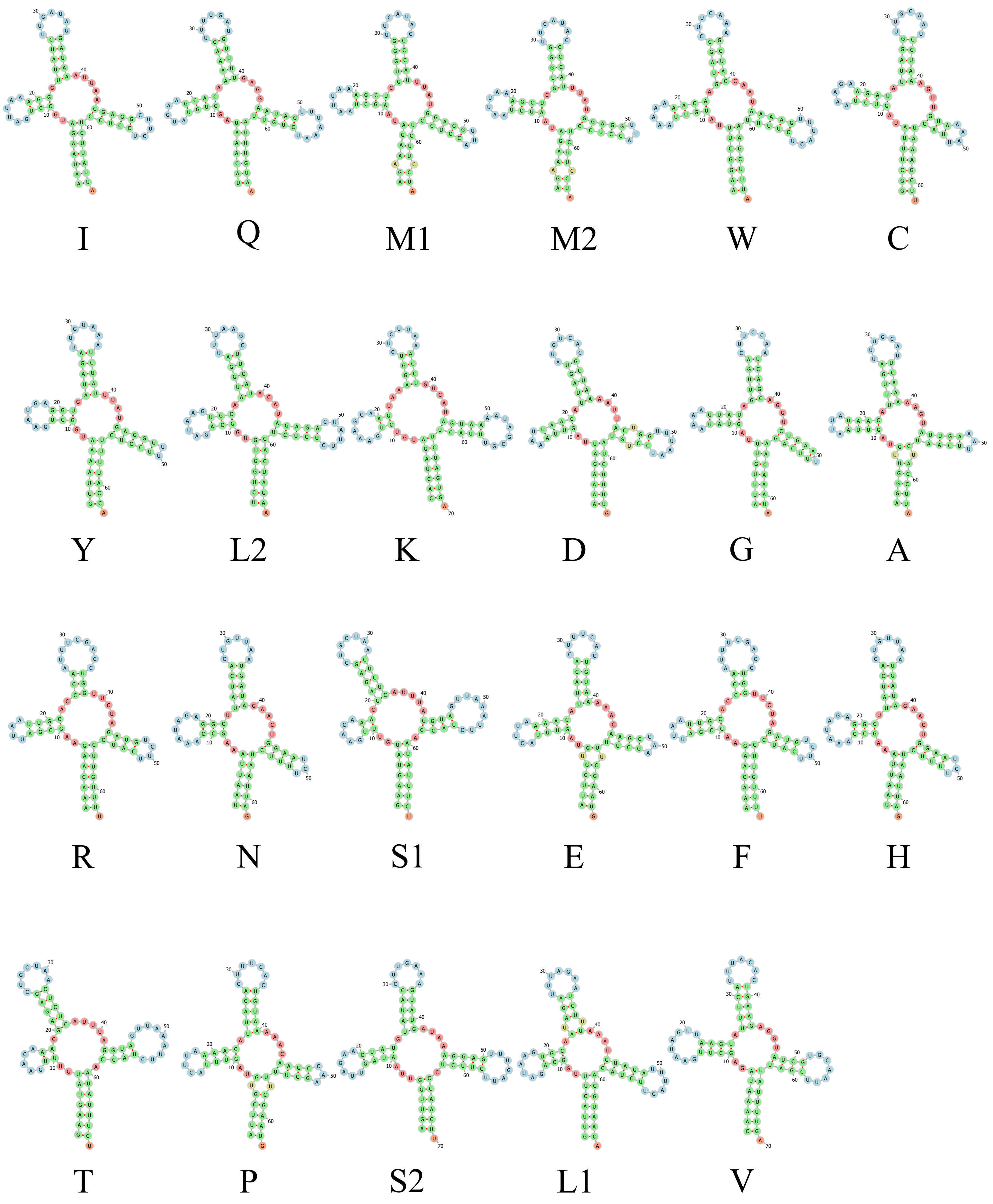

(D) *Ameletus cedrensis*

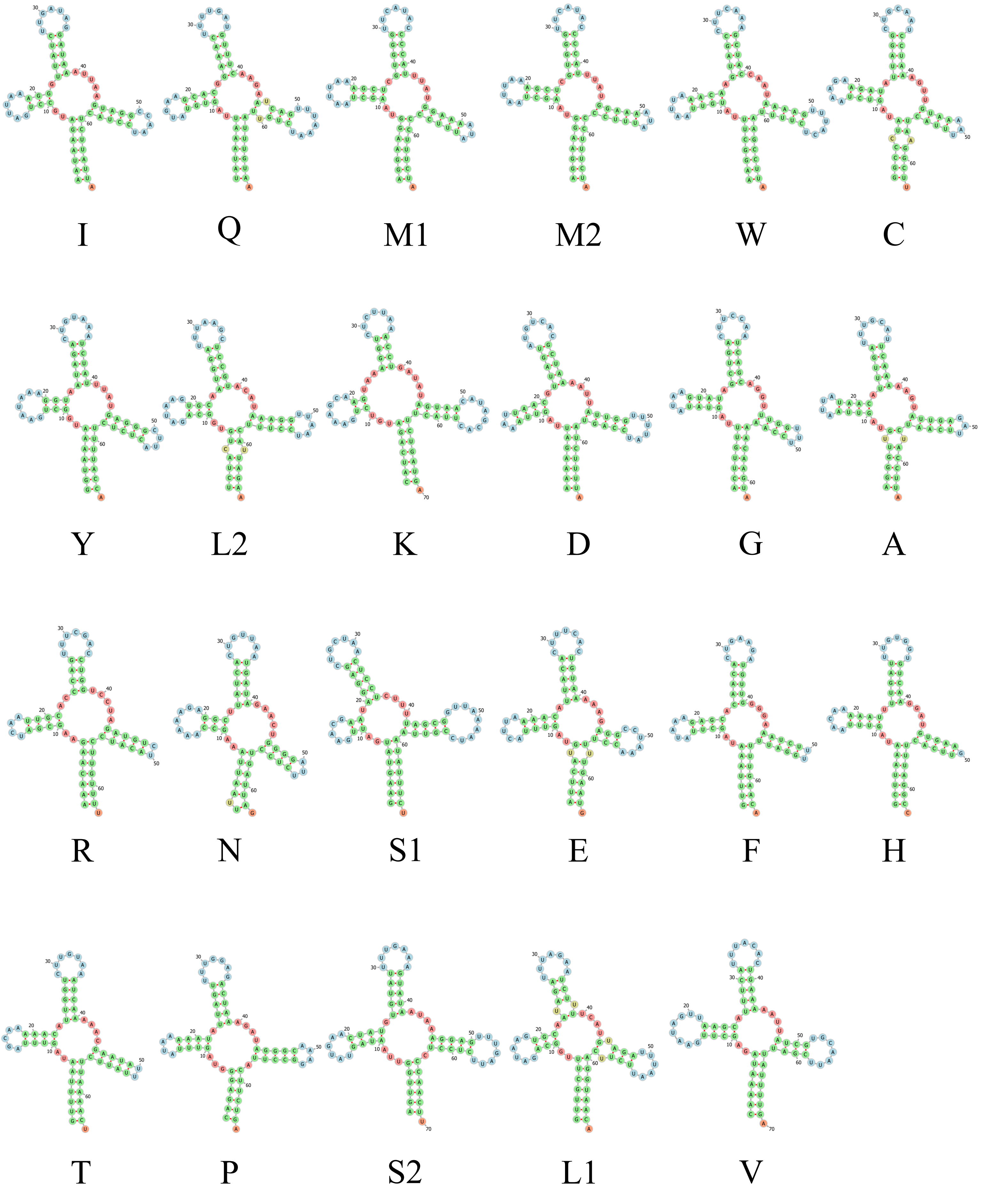

(E) ***Ameletus montanus***

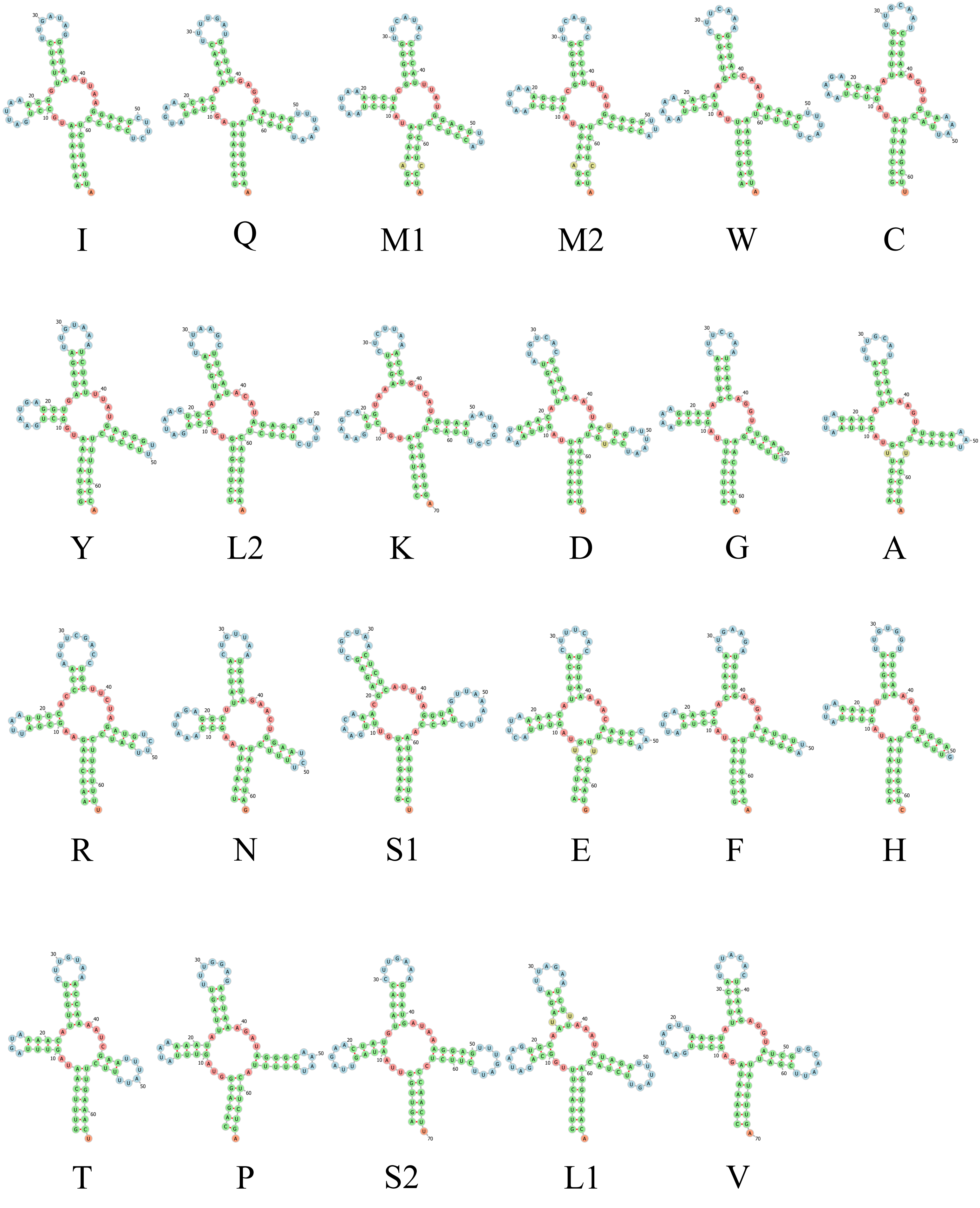

(F) *Ameletus* sp. LNFSFY1

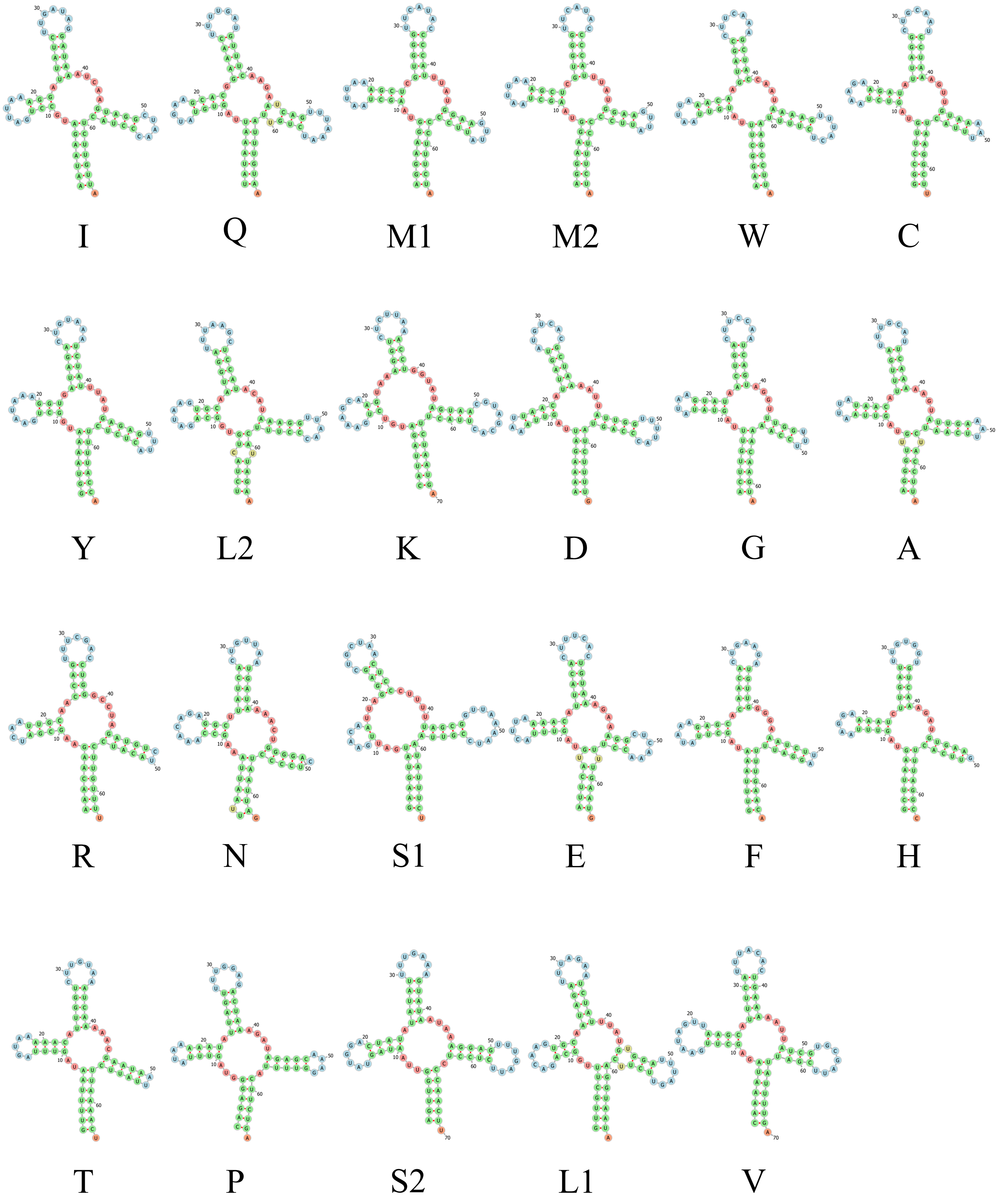

(G) *Ameletus* sp. BZD6

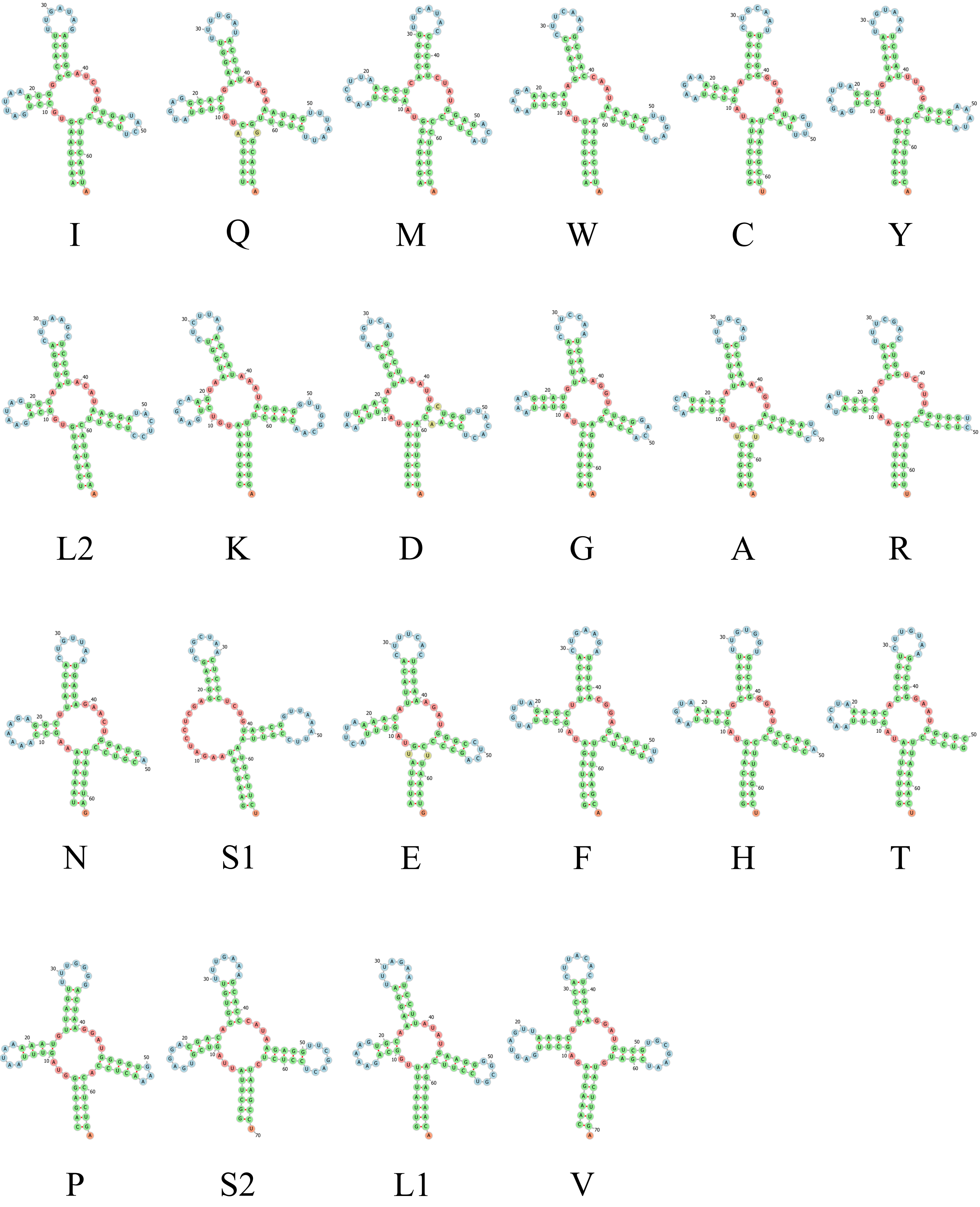

(H) *Isonychia taishunensis*

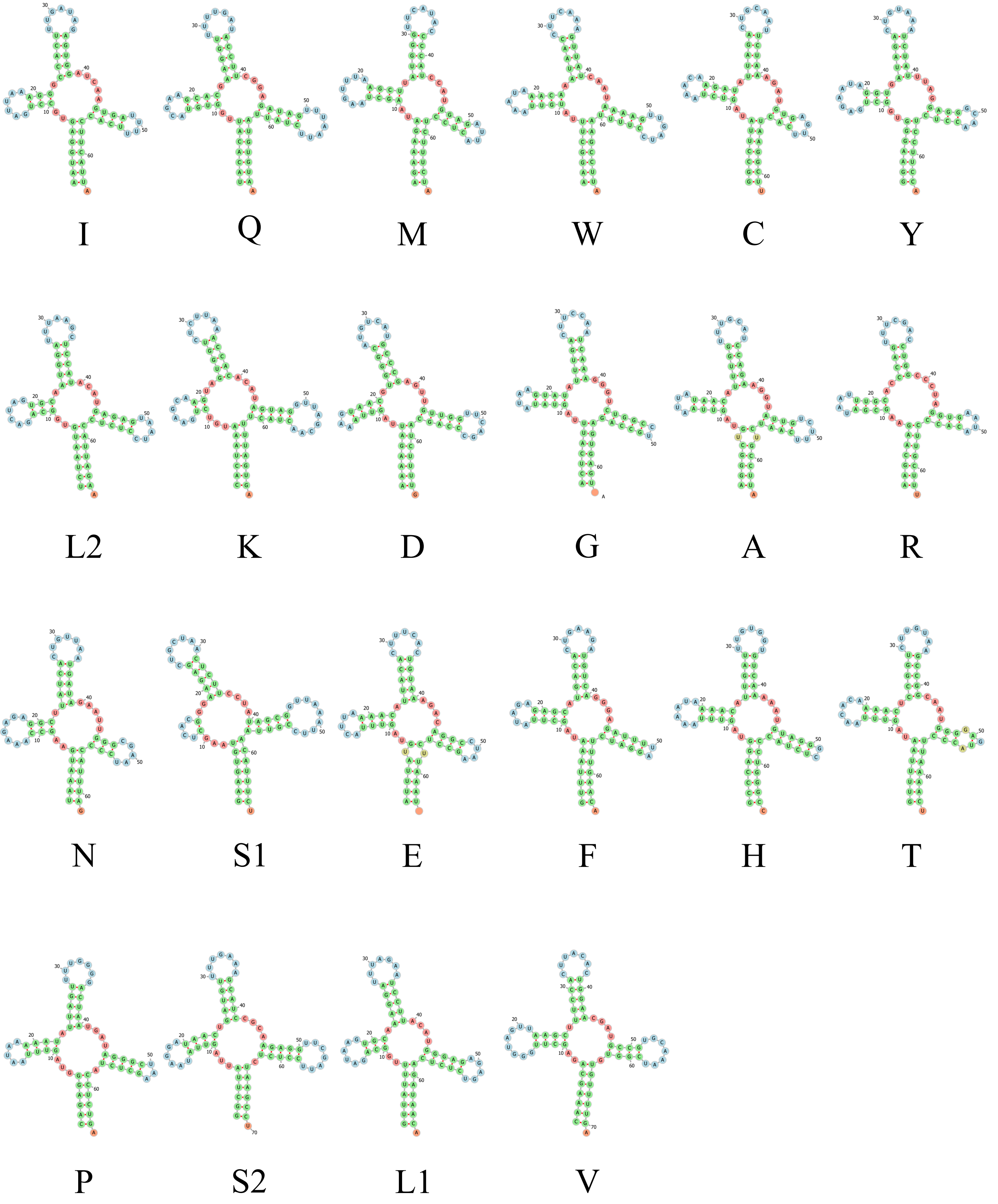

(I) ***Isonychia japonica***

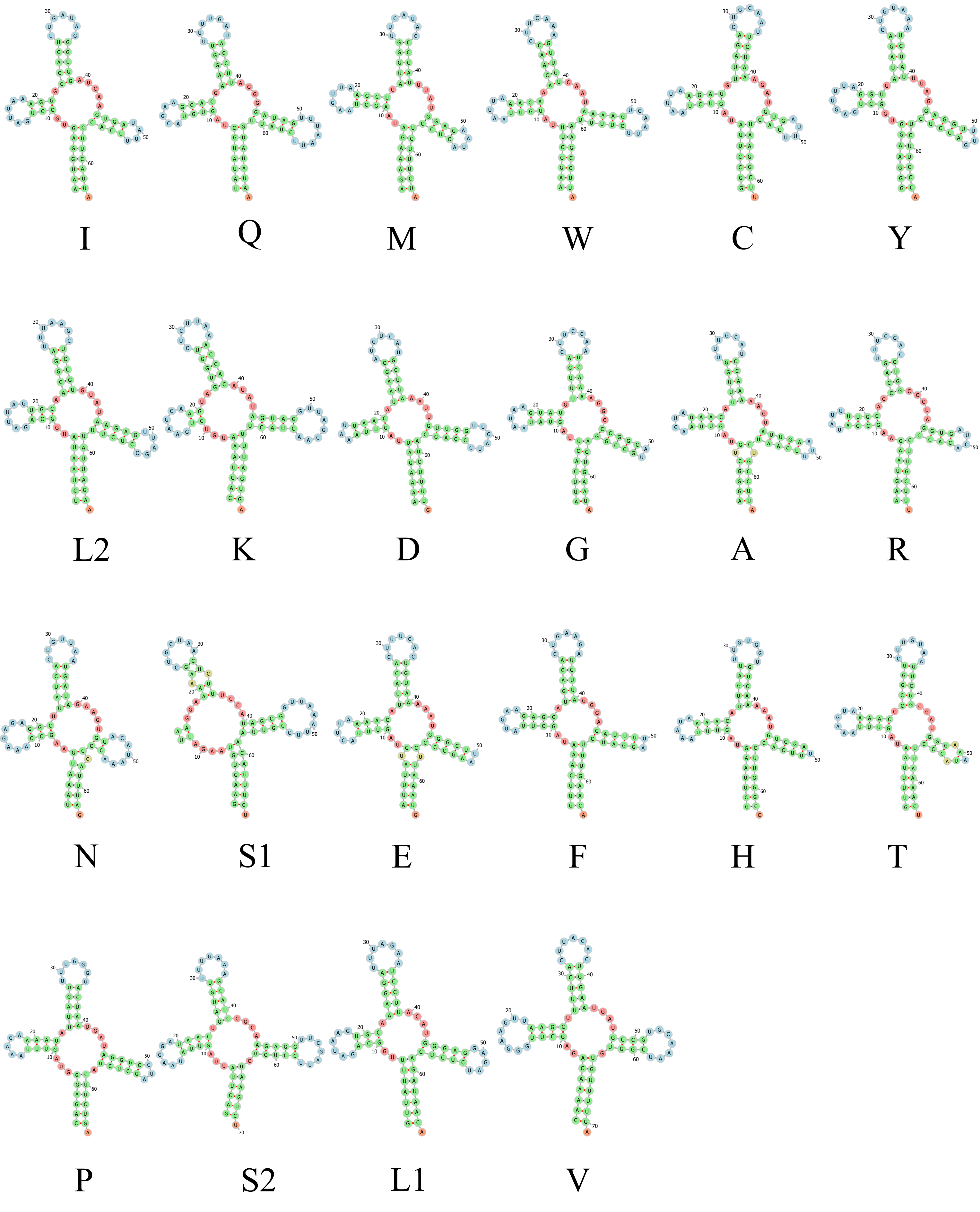

(J) *Isonychia bicolor*

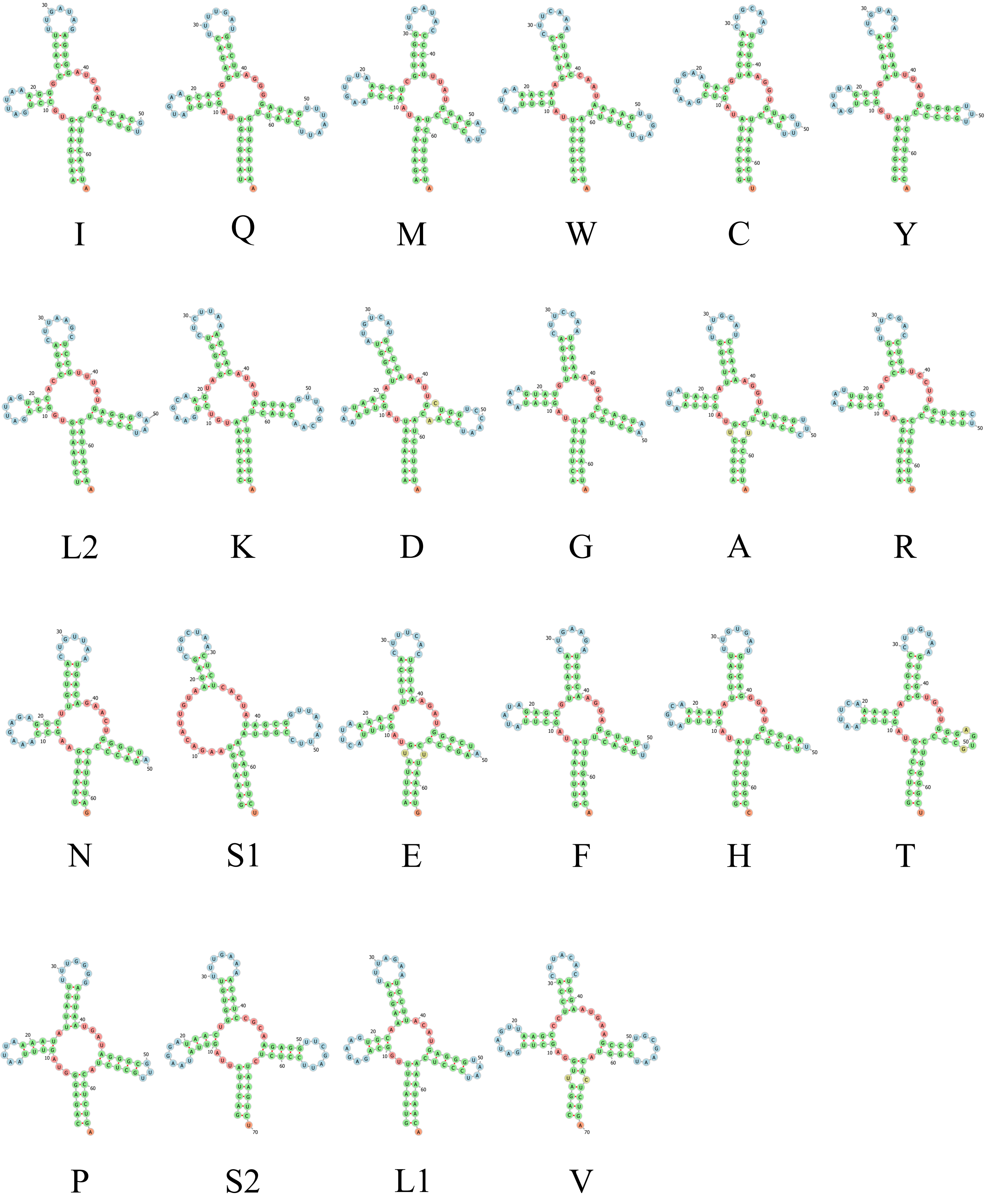

(K) ***Isonychia kiangsinensis*** **02JXDF**

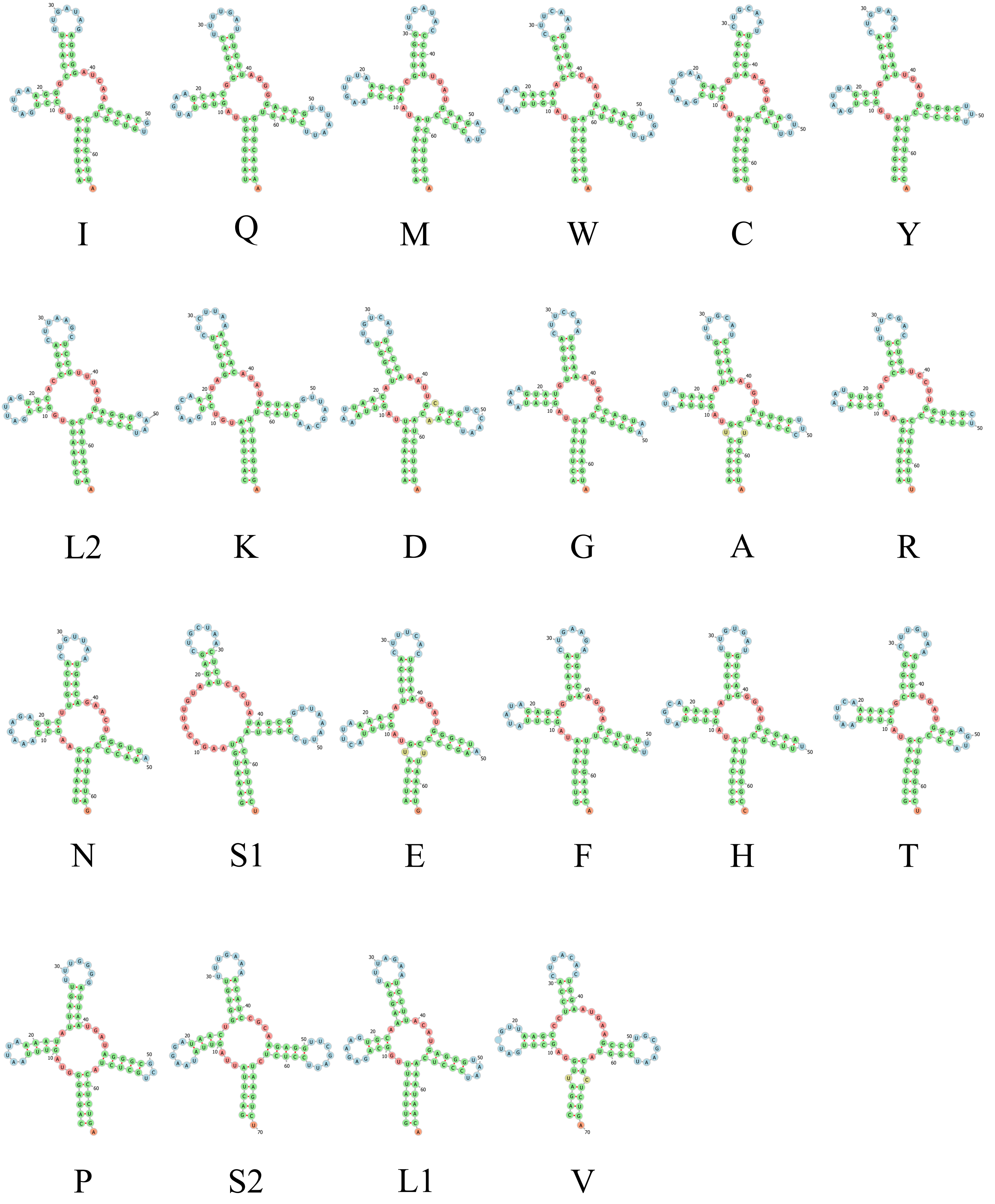

(L) *Isonychia kiangsinensis* 02WZ02

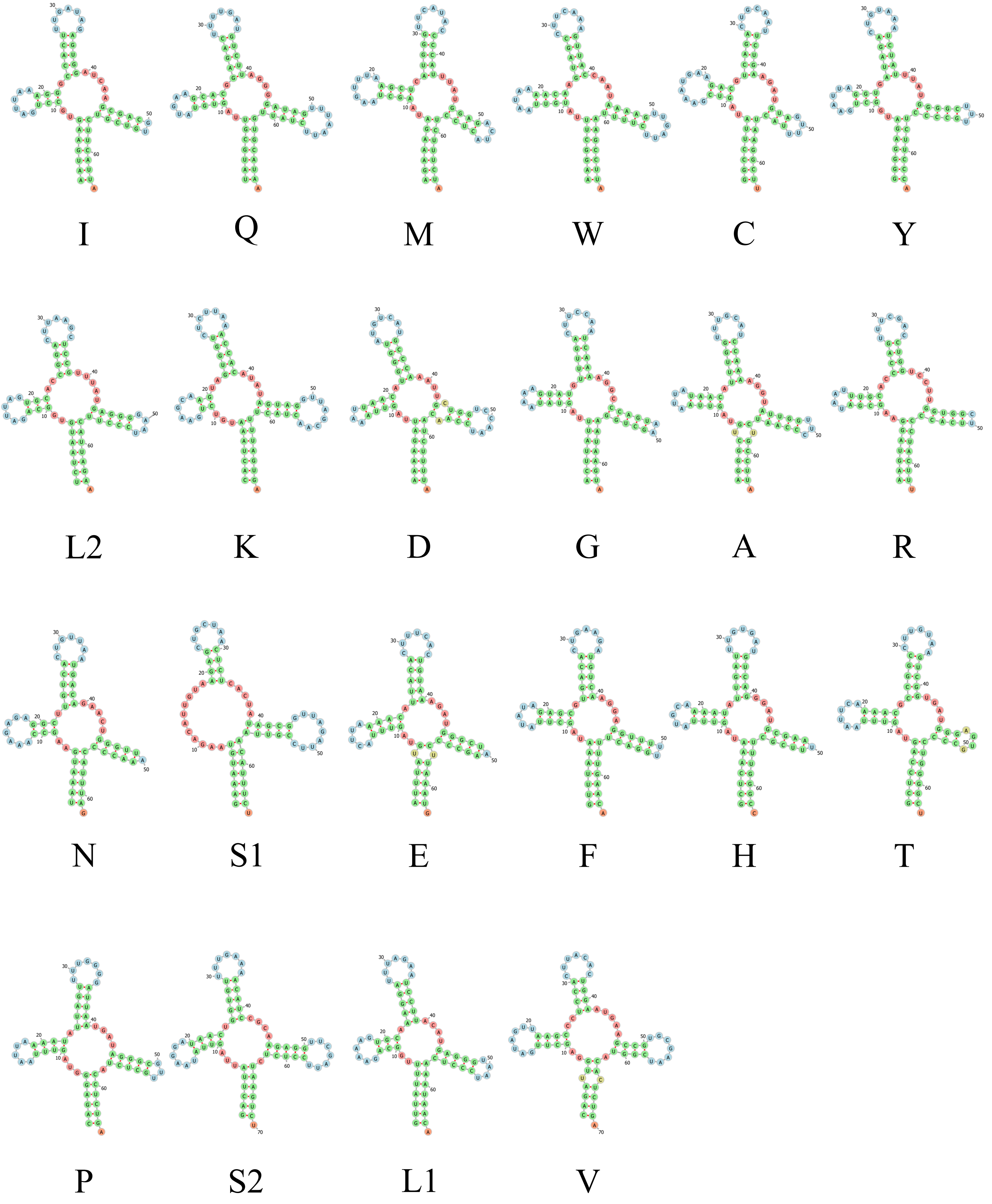

(M) *Isonychia kiangsinensis* 02WZ04

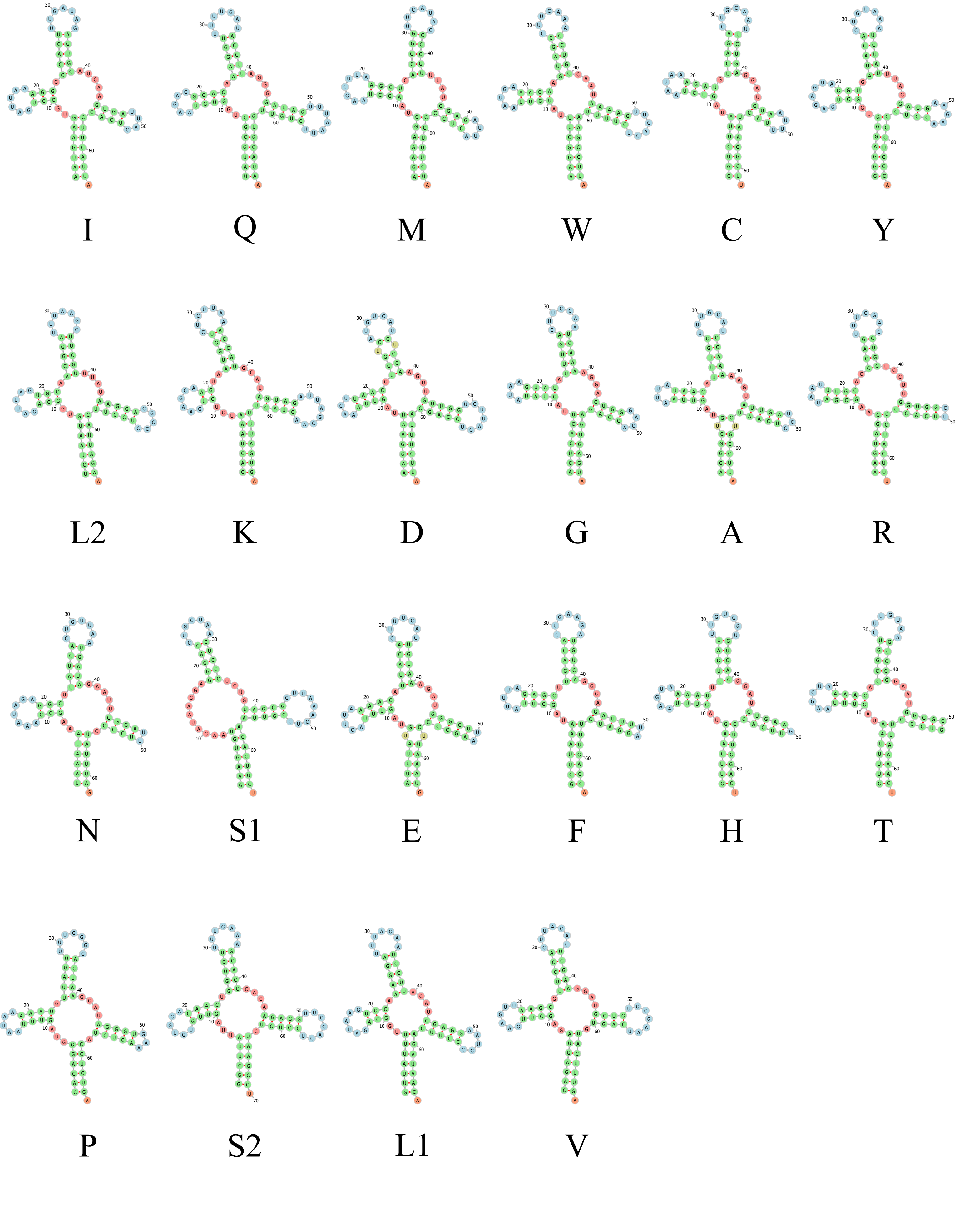

(N) *Isonychia* sp. JLS1

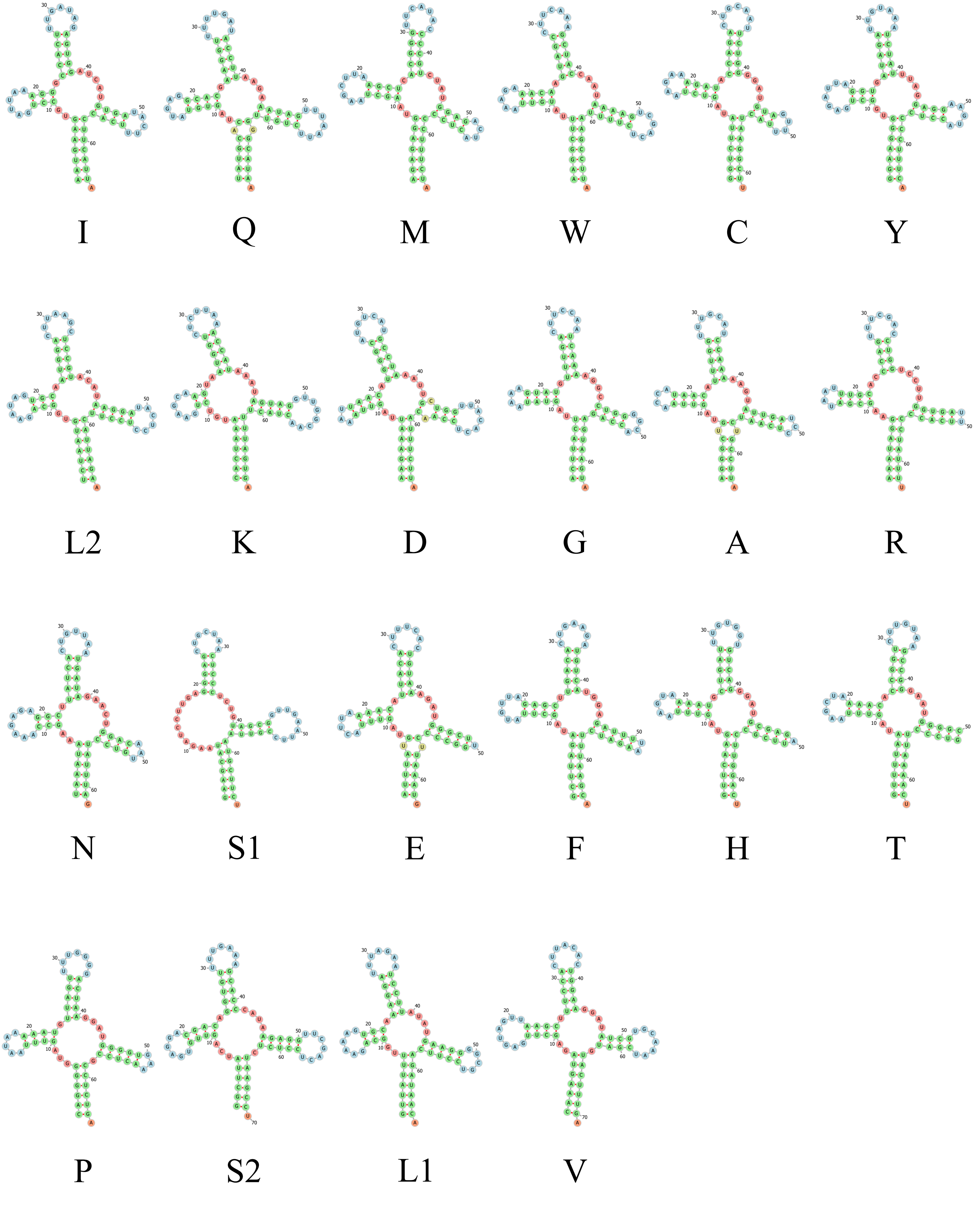

(O) *Isonychia sp.* 02WZ09

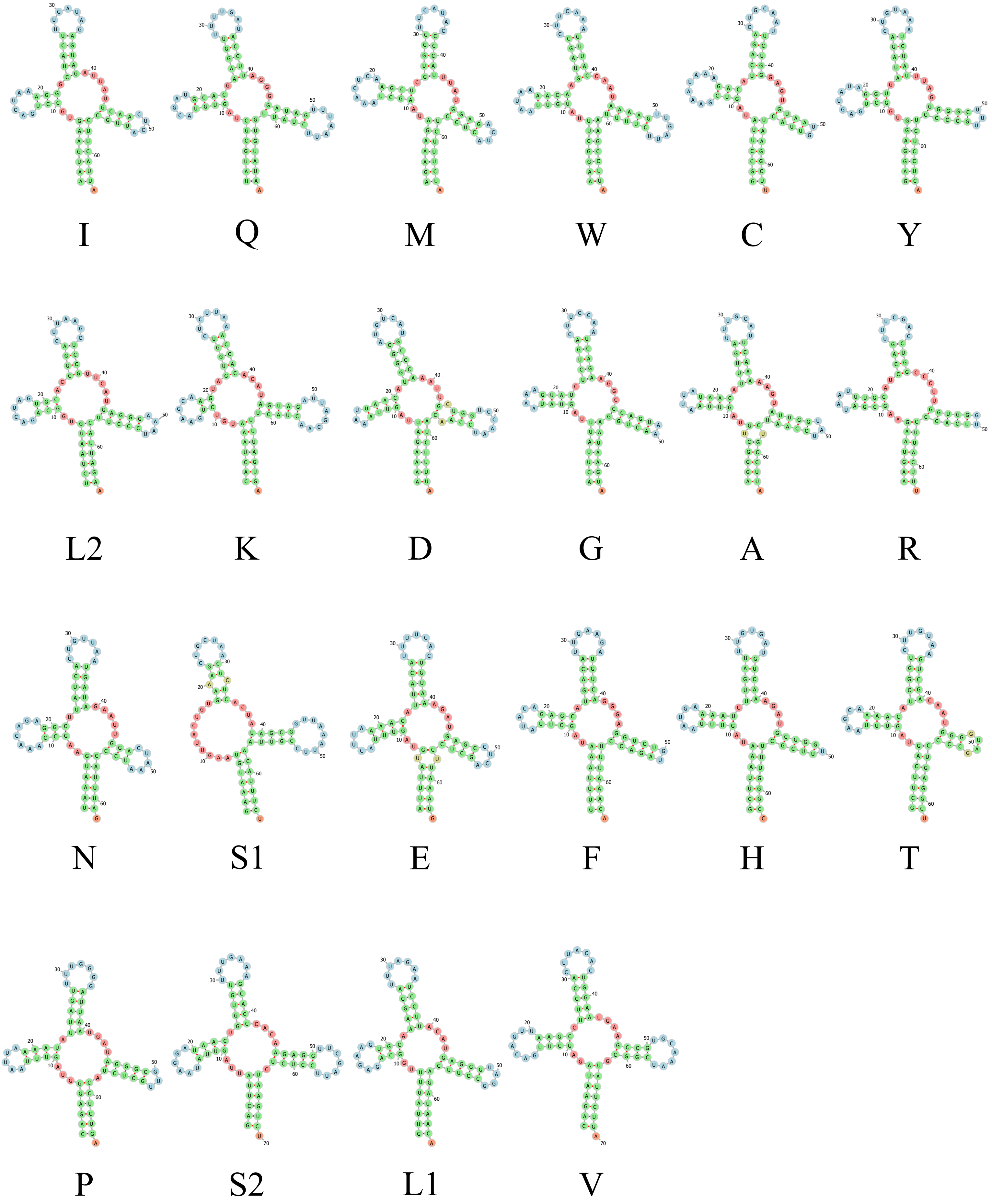

(P) *Isonychia* sp. 9GZST
