# Supplementary material for: Comparative Mitogenomics Reveals Gene Rearrangement and Phylogenetic Relationships in Siphlonuroidea (Insecta: Ephemeroptera)
Source: Insects. 2026 Jul 11;17(7):718. doi: 10.3390/insects17070718 (PMC13410250; doi:10.3390/insects17070718)
Supplement: Supplementary file 1 [file insects-17-00718-s001.zip › Table S1.pdf]

Table S1. Species used in this study for phylogenetic reconstruction and their NCBI GenBank accession numbers.

Order	Family	Species	Length (bp)	GenBank No.	Reference
Archaeognatha	Machilidae	*Coreamachilis coreanus*	15578	MW752137	[74]
Archaeognatha	Machilidae	*Coreamachilis songi*	15570	MW752138	[74]
Archaeognatha	Machilidae	*Petrobius brevistylis*	15698	AY956355	[73]
Archaeognatha	Machilidae	*Pedetontus silvestrii*	15879	EU621793	[71]
]Archaeognatha	Machilidae	*Petrobiellus* sp. 1 JZ-2014	15843	KJ754503	[75]
Archaeognatha	Machilidae	*Trigoniophthalmus alternatus*	16197	EU016193	[72]
Zygentoma	Nicoletiidae	*Atelura formicaria*	15205	EU084035	[76]
Zygentoma	Lepismatidae	*Ctenolepisma longicaudatum*	15242	OQ536441	Unpublished
Zygentoma	Lepismatidae	*Ctenolepisma villosum*	15488	MK301436	[77]
Zygentoma	Lepismatidae	*Thermobia domestica*	15152	AY639935	[78]
Odonata	Aeshnidae	*Anax imperator*	16087	KX161841	[79]
Odonata	Calopterygidae	*Hetaerina titia*	17701	OQ363879	[80]
Odonata	Calopterygidae	*Vestalis melania*	16685	JX050224	[77]
Odonata	Chlorogomphidae	*Chlorogomphus shanicus*	15497	OP572413	[82]
Odonata	Cordulegastridae	*Cordulegaster boltonii*	15384	MT874487	Unpublished
Odonata	Corduliidae	*Epophthalmia elegans*	15719	MK522522	[83]
Odonata	Epiophlebiidae	*Epiophlebia superstes*	15435	JX050223	[84]
Odonata	Gomphidae	*Ictinogomphus* sp. MT-2014	15393	KM244673	[52]
Odonata	Megapodagrionidae	*Mesopodagrion tibetanum*	15363	MK951671	[62]
Odonata	Polythoridae	*Chalcopteryx rutilans*	15653	OQ868370	[85]
Plecoptera	Leuctridae	*Perlomyia kappa*	15759	OQ612623	[86]
Plecoptera	Leuctridae	*Rhopalopsole subnigra*	15562	OQ612622	[86]
Plecoptera	Nemouridae	*Nemurella pictetii*	15934	OR601702	[87]
Plecoptera	Nemouridae	*Ostrocerca truncata*	15971	OR398225	[87]
Plecoptera	Perlidae	*Claassenia magna*	15774	OK012602	[88]
Plecoptera	Perlidae	*Claassenia xucheni*	15777	OK021653	[88]
Plecoptera	Perlodidae	*Filchneria zhouchangfai*	16032	OP414496	Unpublished
Plecoptera	Perlodidae	*Perlodinella shennongjia*	17612	OP414495	Unpublished
Plecoptera	Taeniopterygidae	*Mesyatsia karakorum*	15596	OP414494	Unpublished
Plecoptera	Scopuridae	*Scopura montana*	15966	OQ612621	[86]
Mantodea	Mantidae	*Tamolanica tamolana*	16055	DQ241797	Unpublished
Mantodea	Leptomantellidae	*Leptomantella albella*	15534	KJ463364	[89]
Blattodea	Blattellidae	*Blattella germanica*	15025	EU854321	[90]
Blattodea	Corydiidae	*Eupolyphaga sinensis*	15553	FJ830540	[91]
Coleoptera	Elateridae	*Pyrophorus divergens*	16120	EF398270	[93]
Coleoptera	Chaetosomatidae	*Chaetosoma scaritides*	15511	EU877951	[92]
Ephemeroptera	Ameletidae	*Ameletus* sp. 1 MT-2014	15141	KM244682	[52]
Ephemeroptera	Ameletidae	*Ameletus cedrensis*	16454	PX943394	This study
Ephemeroptera	Ameletidae	*Ameletus montanus*	16671	PX943393	This study
Ephemeroptera	Ameletidae	*Ameletus* sp. LNFSFY1	16687	PX943391	This study
Ephemeroptera	Ameletidae	*Ameletus* sp. BZD6	15280	PX943392	This study
Ephemeroptera	Baetidae	*Baetis atlanticus*	17607	OZ373116	Unpublished
Ephemeroptera	Baetidae	*Baetis rhodani*	16901	OZ391886	Unpublished
Ephemeroptera	Baetidae	*Baetis* sp. PC-2010	14883	GU936204	[61]
Ephemeroptera	Baetidae	*Cloeon dipterum*	15407	MW149047	[61]
Ephemeroptera	Baetidae	*Cloeon viridulum*	14431	PQ064108	[58]

Ephemeroptera	Baetidae	*Nigrobaetis niger*	16372	MT483692	Unpublished
Ephemeroptera	Baetidae	*Procloeon bifidum*	15130	MT483677	Unpublished
Ephemeroptera	Baetidae	*Takobia yixiani*	14589	GU479735	[61]
Ephemeroptera	Behningiidae	*Behningia nujiangensis*	15459	OQ439817	Unpublished
Ephemeroptera	Caenidae	*Caenis horaria*	15479	MT622520	Unpublished
Ephemeroptera	Caenidae	*Caenis robusta*	15855	MT584126	Unpublished
Ephemeroptera	Caenidae	*Caenis* sp. JYZ-2020	15392	MN356096	[65]
Ephemeroptera	Ephemerellidae	*Cincticostella fusca*	15135	MT535767	[36]
Ephemeroptera	Ephemerellidae	*Cincticostella femorata*	15594	PX508693	[66]
Ephemeroptera	Ephemerellidae	*Cincticostella gosei*	15416	PX508691	[66]
Ephemeroptera	Ephemerellidae	*Drunella ishiyamana*	16485	PX508690	[66]
Ephemeroptera	Ephemerellidae	*Ephemerella* sp. Yunnan-2018	15256	MT274127	[37]
Ephemeroptera	Ephemerellidae	*Serratella ignita*	14772	MT628582	Unpublished
Ephemeroptera	Ephemerellidae	*Serratella* sp. Liaoning-2019	15523	MT274128	[37]
Ephemeroptera	Ephemerellidae	*Serratella zapekinae*	15703	MT274130	[37]
Ephemeroptera	Ephemerellidae	*Spinorea montana*	16219	PX508689	Unpublished
Ephemeroptera	Ephemerellidae	*Teloganopsis jinghongensis*	15626	PX508687	[66]
Ephemeroptera	Ephemerellidae	*Torleya grandiforceps*	15330	MT274131	[37]
Ephemeroptera	Ephemerellidae	*Torleya mikhaili*	15042	MT535766	[36]
Ephemeroptera	Ephemerellidae	*Torleya nepalica*	15599	MT274132	[37]
Ephemeroptera	Ephemerellidae	*Uracanthella punctisetae*	15435	PX508692	[66]
Ephemeroptera	Ephemeridae	*Ephemera danica*	15515	MT483675	Unpublished
Ephemeroptera	Ephemeridae	*Ephemera orientalis*	16463	EU591678	[70]
Ephemeroptera	Ephemeridae	*Ephemera pieli*	15579	OQ439822	Unpublished
Ephemeroptera	Ephemeridae	*Ephemera serica*	15004	OK018134	[64]
Ephemeroptera	Ephemeridae	*Ephemera shengmi*	15149	MF352161	[13]
Ephemeroptera	Ephemeridae	*Ephemera* sp. XL-2019	15314	MK951659	[62]
Ephemeroptera	Ephemeridae	*Ephemera vulgata*	15734	MT483620	Unpublished
Ephemeroptera	Ephemeridae	*Hexagenia rigida*	16159	OL678102	[63]
Ephemeroptera	Euthyplociidae	*Polyplocia orientalis*	15251	OQ439819	Unpublished
Ephemeroptera	Heptageniidae	*Afronurus rubromaculatus*	15519	MK642294	[35]
Ephemeroptera	Heptageniidae	*Afronurus* sp. 'furcata'	15334	MW381292	[55]
Ephemeroptera	Heptageniidae	*Afronurus yixingensis*	15883	MK642297	[35]
Ephemeroptera	Heptageniidae	*Cinygmina obliquistrita*	15532	MN938925	Unpublished
Ephemeroptera	Heptageniidae	*Cinygmina* sp. 1 YW01BF06	15360	MK642295	[35]
Ephemeroptera	Heptageniidae	*Ecdyonurus torrentis*	15686	OX439138	[53]
Ephemeroptera	Heptageniidae	*Ecdyonurus* sp. LNTH142	15453	PP576365	[21]
Ephemeroptera	Heptageniidae	*Electrogena lateralis*	15378	MT874480	Unpublished
Ephemeroptera	Heptageniidae	*Epeorus aculeatus*	15451	OK495695	[59]
Ephemeroptera	Heptageniidae	*Epeorus alexandri*	15836	OK495699	[59]
Ephemeroptera	Heptageniidae	*Epeorus bifurcatus*	15467	MW381293	[55]
Ephemeroptera	Heptageniidae	*Epeorus bispinosus*	15452	OK495696	[59]
Ephemeroptera	Heptageniidae	*Epeorus carinatus*	15338	MT112896	[69]
Ephemeroptera	Heptageniidae	*Epeorus dayongensis*	15509	OK495703	[59]
Ephemeroptera	Heptageniidae	*Epeorus gibbus*	15839	OK495692	[59]
Ephemeroptera	Heptageniidae	*Epeorus herklotsi*	15508	OK495701	[59]
Ephemeroptera	Heptageniidae	*Epeorus melli*	15490	MW381294	[55]
Ephemeroptera	Heptageniidae	*Epeorus montanus*	15472	MW381295	[55]
Ephemeroptera	Heptageniidae	*Epeorus pellucidus*	15435	MW381296	[55]

Ephemeroptera	Heptageniidae	*Epeorus psi*	15654	OK495704	[59]
Ephemeroptera	Heptageniidae	*Epeorus rhithralis*	15447	OK495697	[59]
Ephemeroptera	Heptageniidae	*Epeorus sinensis*	15508	PP526256	[21]
Ephemeroptera	Heptageniidae	*Epeorus* sp. LA03FY06	15514	MK642299	[35]
Ephemeroptera	Heptageniidae	*Epeorus* sp. MT-2014	15456	KM244708	[52]
Ephemeroptera	Heptageniidae	*Epeorus* sp. 01 ZXM-2022a	15498	OK495694	[59]
Ephemeroptera	Heptageniidae	*Epeorus unispinosus*	15849	OK495693	[59]
Ephemeroptera	Heptageniidae	*Heptagenia sulphurea*	15337	MT872698	Unpublished
Ephemeroptera	Heptageniidae	*Leucrocuta aphrodite*	15428	MK642301	[35]
Ephemeroptera	Heptageniidae	*Maccaffertium mediopunctatum*	15324	MK642303	[35]
Ephemeroptera	Heptageniidae	*Maccaffertium vicarium*	15324	MK642304	[35]
Ephemeroptera	Heptageniidae	*Notacanthurus lamellosus*	15693	MW381298	[55]
Ephemeroptera	Heptageniidae	*Notacanthurus* sp. 'maculosus'	15524	MW381299	[55]
Ephemeroptera	Heptageniidae	*Paegniodes cupulatus*	15721	MW381300	[55]
Ephemeroptera	Heptageniidae	*Parafronurus youi*	15481	EU349015	[68]
Ephemeroptera	Heptageniidae	*Parafronurus* sp. 16bf10	15527	PP554243	[21]
Ephemeroptera	Heptageniidae	*Rhithrogena germanica*	15251	MT584121	Unpublished
Ephemeroptera	Heptageniidae	*Stenacron interpunctatum*	15330	MK642305	[35]
Ephemeroptera	Heptageniidae	*Stenonema femoratum*	15332	MK642306	[35]
Ephemeroptera	Isonychiidae	*Isonychia ignota*	15105	HM143892	Unpublished
Ephemeroptera	Isonychiidae	*Isonychia kiangsinensis*	15456	MH119135	[67]
Ephemeroptera	Isonychiidae	*Isonychia* sp. XL-2019	15618	MK951658	[62]
Ephemeroptera	Isonychiidae	*Isonychia taishunensis*	16484	PX943383	This study
Ephemeroptera	Isonychiidae	*Isonychia valida*	15566	PX943382	This study
Ephemeroptera	Isonychiidae	*Isonychia bicolor*	15084	PX943390	This study
Ephemeroptera	Isonychiidae	*Isonychia kiangsinensis* 02JXDF	15107	PX943389	This study
Ephemeroptera	Isonychiidae	*Isonychia kiangsinensis* 02WZ02	15597	PX943388	This study
Ephemeroptera	Isonychiidae	*Isonychia kiangsinensis* 02WZ04	15592	PX943387	This study
Ephemeroptera	Isonychiidae	*Isonychia* sp. JLS1	15960	PX943384	This study
Ephemeroptera	Isonychiidae	*Isonychia* sp. 02WZ09	15342	PX943386	This study
Ephemeroptera	Isonychiidae	*Isonychia* sp. 9GZST	15453	PX943385	This study
Ephemeroptera	Leptophlebiidae	*Choroterpes yixingensis*	15534	MW717290	[54]
Ephemeroptera	Leptophlebiidae	*Deleatidium vernale*	15511	OR414023	Unpublished
Ephemeroptera	Leptophlebiidae	*Leptophlebia marginata*	15051	MT622514	Unpublished
Ephemeroptera	Leptophlebiidae	*Leptophlebia vespertina*	14870	MT622515	Unpublished
Ephemeroptera	Leptophlebiidae	*Paraleptophlebia submarginata*	15361	MT872692	Unpublished
Ephemeroptera	Neoephemeridae	*Pulchephemera projecta*	16031	OK272542	[56]
Ephemeroptera	Neoephemeridae	*Potamanthellus edmundsi*	15274	OK272543	[56]
Ephemeroptera	Palingeniidae	*Anagenesia paradoxa*	15163	OQ439821	Unpublished
Ephemeroptera	Polymitarcyidae	*Ephoron nanchangi*	14997	OQ439818	Unpublished
Ephemeroptera	Potamanthidae	*Potamanthus longitibius*	15761	PP473794	[34]
Ephemeroptera	Potamanthidae	*Potamanthus sangangensis*	15946	OQ450153	Unpublished
Ephemeroptera	Potamanthidae	*Potamanthus* sp. 02JHGD	17119	PP473796	[34]
Ephemeroptera	Potamanthidae	*Potamanthus* sp. 08HH02	15859	PP473798	[34]
Ephemeroptera	Potamanthidae	*Rhoenanthus coreanus*	15480	PP473799	[34]
Ephemeroptera	Potamanthidae	*Rhoenanthus obscurus*	14968	PP473793	[34]
Ephemeroptera	Potamanthidae	*Rhoenanthus youi*	15232	PQ723067	[57]
Ephemeroptera	Siphlonuridae	*Siphlonurus aestivalis*	15120	MT862395	Unpublished
Ephemeroptera	Siphlonuridae	*Siphlonurus alternatus*	19363	OX463789	Unpublished

Ephemeroptera	Siphlonuridae	*Siphlonurus chankae*	16220	PV534073	[60]
Ephemeroptera	Siphlonuridae	*Siphlonurus davidi*	15667	PV557747	[60]
Ephemeroptera	Siphlonuridae	*Siphlonurus dongxi*	16035	PV557748	[60]
Ephemeroptera	Siphlonuridae	*Siphlonurus immanis*	15598	PV534074	[60]
Ephemeroptera	Siphlonuridae	*Siphlonurus immanis*	16138	PX943381	This study
Ephemeroptera	Siphlonuridae	*Siphlonurus lacustris*	15496	PV557749	[60]
Ephemeroptera	Siphlonuridae	*Siphlonurus palaearcticus*	16060	PV534075	[60]
Ephemeroptera	Siphlonuridae	*Siphlonurus zhelochovtsevi*	15115	PV557752	[60]
Ephemeroptera	Siphlonuridae	*Siphlonurus zhelochovtsevi*	16006	PX943379	This study
Ephemeroptera	Siphlonuridae	*Siphlonurus* sp. FJND2	15939	PX943380	This study
Ephemeroptera	Siphlonuridae	*Siphlonurus* sp. 1 XHQ-2025a	14912	PV557750	[60]
Ephemeroptera	Siphlonuridae	*Siphlonurus* sp. 2 XHQ-2025a	15213	PV557751	[60]
Ephemeroptera	Siphlonuridae	*Siphlonurus* sp. MT-2014	14745	KM244684	[52]
Ephemeroptera	Siphluriscidae	*Siphluriscus chinensis*	16616	HQ875717	[14]
Ephemeroptera	Siphluriscidae	*Siphluriscus* sp. l JZ-2022	15212	ON729391	[33]
Ephemeroptera	Teloganodidae	*Teloganodidae* sp. MT-2014	12435	KM244703	[52]
Ephemeroptera	Vietnamellidae	*Vietnamella sinensis isolate* tl	15674	OK265109	[20]
Ephemeroptera	Vietnamellidae	*Vietnamella sinensis isolate* qy	15610	OK265110	[20]
Ephemeroptera	Vietnamellidae	*Vietnamella sinensis isolate* cn	15674	OK265111	[20]
Ephemeroptera	Vietnamellidae	*Vietnamella* sp. MT-2014	15043	KM244655	[52]
