# Supplementary material for: Comparative Mitogenomics Reveals Gene Rearrangement and Phylogenetic Relationships in Siphlonuroidea (Insecta: Ephemeroptera)
Source: Insects. 2026 Jul 11;17(7):718. doi: 10.3390/insects17070718 (PMC13410250; doi:10.3390/insects17070718)
Supplement: Supplementary file 1 [file insects-17-00718-s001.zip › Table S2.pdf]

Table S2. The results of codon saturation analysis for the first, second, and third codon positions of protein coding genes in 164 mitochondrial genomes.

Codon position	Iss	Iss.c		*p*
		Sym	Asym	
codon 1	0.293	0.806	0.546	0.000
codon 2	0.146	0.806	0.546	0.000
codon 3	0.752	0.806	0.546	0.000
