# Supplementary material for: Comparative Mitogenomics Reveals Gene Rearrangement and Phylogenetic Relationships in Siphlonuroidea (Insecta: Ephemeroptera)
Source: Insects. 2026 Jul 11;17(7):718. doi: 10.3390/insects17070718 (PMC13410250; doi:10.3390/insects17070718)
Supplement: Supplementary file 1 [file insects-17-00718-s001.zip › Table S3.pdf]

Table S3. Best partitioning schemes and best evolutionary models selected by PartitionFinder v.2.1.1.

Nucleotide sequence alignments		
**Subset**	**Subset partitions**	**Best model**
Partition 1	*ATP6*_codon1	GTR+I+G
Partition 2	*Cyt b*_codon2, *COX2*_codon2, *COX3*_codon2, *ATP6*_codon2	TVM+I+G
Partition 3	*ND2*_codon1, *ATP8*_codon1	GTR+I+G
Partition 4	*ATP8*_codon2, *ND3*_codon2, *ND2*_codon2, *ND6*_codon2	GTR+I+G
Partition 5	*COX1*_codon1	GTR+I+G
Partition 6	*COX1*_codon2	TVM+I+G
Partition 7	*COX2*_codon1, *COX3*_codon1	GTR+I+G
Partition 8	*Cyt b*_codon1	GTR+I+G
Partition 9	*ND4*_codon1, *ND4L*_codon1, *ND1*_codon1	GTR+I+G
Partition 10	*ND4L*_codon2, *ND5*_codon2, *ND1*_codon2, *ND4*_codon2	GTR+I+G
Partition 11	*ND6*_codon1, *ND3*_codon1	GTR+I+G
Partition 12	*ND1*_codon1	GTR+I+G
