# Supplementary material for: Comparative Mitogenomics Reveals Gene Rearrangement and Phylogenetic Relationships in Siphlonuroidea (Insecta: Ephemeroptera)
Source: Insects. 2026 Jul 11;17(7):718. doi: 10.3390/insects17070718 (PMC13410250; doi:10.3390/insects17070718)
Supplement: Supplementary file 1 [file insects-17-00718-s001.zip › Table S4.pdf]

Table S4. Best partitioning schemes and best evolutionary models selected by ModelFinder.

Nucleotide sequence alignments		
**Subset**	**Subset partitions**	**Best model**
Partition 1	*ATP6*_codon1	GTR+F+I+R5
Partition 2	*ATP6*_codon2, *COX2*_codon2, *COX3*_codon2, *Cyt b*_codon2	TVM+F+R4
Partition 3	*ATP8*_codon1, *ND2*_codon1	GTR+F+I+R6
Partition 4	*ATP8*_codon2	GTR+F+G4
Partition 5	*COX1*_codon1	GTR+F+I+G4
Partition 6	*COX1*_codon2	TVM+F+I+R3
Partition 7	*COX2*_codon1, *COX3*_codon1	GTR+F+I+R5
Partition 8	*Cyt b*_codon1	GTR+F+I+R6
Partition 9	*ND1*_codon1, *ND4L*_codon1, *ND4*_codon1, *ND5*_codon1	GTR+F+I+R6
Partition 10	*ND1*_codon2, *ND4L*_codon2, *ND4*_codon2, *ND5*_codon2	GTR+F+R6
Partition 11	*ND2*_codon2, *ND3*_codon2, *ND6*_codon2	GTR+F+R5
Partition 12	*ND3*_codon1, *ND6*_codon1	GTR+F+R6
