# Supplementary material for: Comparative Mitogenomics Reveals Gene Rearrangement and Phylogenetic Relationships in Siphlonuroidea (Insecta: Ephemeroptera)
Source: Insects. 2026 Jul 11;17(7):718. doi: 10.3390/insects17070718 (PMC13410250; doi:10.3390/insects17070718)
Supplement: Supplementary file 1 [file insects-17-00718-s001.zip › Table S5.pdf]

Table S5. Location of features of the 16 newly sequenced mitogenomes.

A. *Siphlonurus immanis* HLJFY2

Gene	Strand	Size	Position		Codon	
			From	To	Start	Stop
trnI	H	67	1	67		
trnQ	L	61	56	116		
trnM	H	66	116	181		
Q_copy2	L	69	330	398		
M_copy2	H	66	398	463		
Q_copy3	L	69	612	680		
M_copy3	H	66	680	745		
Q_copy4	L	69	894	962		
M_copy4	H	66	962	1027		
ND2	H	1035	1028	2062	GTG	TAA
trnW	H	68	2061	2128		
trnC	L	63	2121	2183		
trnY	L	66	2184	2249		
COX1	H	1540	2242	3781	ATT	T
trnL	H	66	3782	3847		
COX2	H	688	3853	4540	ATG	T
trnK	H	70	4541	4610		
trnD	H	66	4610	4675		
ATP8	H	159	4676	4834	ATC	TAA
ATP6	H	675	4831	5505	ATA	TAA
COX3	H	789	5505	6293	ATG	TAA
trnG	H	64	6300	6363		
ND3	H	348	6370	6717	ATG	TAG
trnA	H	64	6716	6779		
trnR	H	64	6779	6842		
trnN	H	66	6842	6907		
trnS	H	67	6908	6974		
trnE	H	64	6976	7039		
trnF	L	64	7038	7101		
ND5	L	1735	7102	8836	ATG	T
trnH	L	64	8838	8901		
ND4	L	1347	8901	10247	ATG	TAA
ND4L	L	297	10241	10537	ATG	TAA
trnT	H	64	10540	10603		
trnP	L	65	10605	10669		
ND6	H	507	10690	11196	ATG	TAA
Cyt b	H	1137	11196	12332	ATG	TAA
S_copy2	H	70	12331	12400		
ND1	L	945	12420	13364	ATG	TAA
L_copy2	L	67	13366	13432		
rrnL	L	1297	13433	14729		
trnV	L	71	14730	14800		
rrnS	L	788	14801	15588		

B. *Siphlonurus zhelochovtsevi* JLFY14

Gene	Strand	Size	Position		Codon	
			From	To	Start	Stop
trnI	H	67	1	67		
trnM	H	66	121	186		
trnQ	L	66	262	327		
M_copy2	H	66	327	392		
ND2	H	1035	393	1427	GTG	TAA
trnW	H	68	1426	1493		
trnC	L	63	1486	1548		
trnY	L	67	1549	1615		
COX1	H	1540	1608	3147	ATT	T
trnL	H	66	3148	3213		
COX2	H	688	3220	3907	ATG	T
trnK	H	70	3908	3977		
trnD	H	66	3977	4042		
ATP8	H	159	4043	4201	ATT	TAA
ATP6	H	675	4198	4872	ATA	TAA
COX3	H	789	4872	5660	ATG	TAA
trnG	H	64	5667	5730		
ND3	H	354	5731	6084	ATT	TAG
trnA	H	64	6083	6146		
trnR	H	64	6146	6209		
trnN	H	66	6209	6274		
trnS	H	67	6275	6341		
trnE	H	64	6343	6406		
trnF	L	64	6405	6468		
ND5	L	1723	6481	8203	ATG	T
trnH	L	64	8205	8268		
ND4	L	1347	8268	9614	ATG	TAA
ND4L	L	297	9608	9904	ATG	TAA
trnT	H	64	9907	9970		
trnP	L	65	9972	10036		
ND6	H	513	10051	10563	ATT	TAA
Cyt b	H	1137	10563	11699	ATG	TAA
S_copy2	H	70	11698	11767		
ND1	L	945	11820	12764	ATG	TAA
L_copy2	L	67	12766	12832		
rrnL	L	1297	12833	14129		
trnV	L	71	14130	14200		
rrnS	L	787	14201	14987		

C. *Siphlonurus* sp. FJND2

Gene	Strand	Size	Position		Codon	
			From	To	Start	Stop
trnI	H	67	1	67		
trnQ	L	59	56	114		
trnM	H	67	117	183		
Q_copy2	L	69	255	323		
M_copy2	H	67	326	392		
Q_copy3	L	69	464	532		
M_copy3	H	67	535	601		
Q_copy4	L	69	673	741		
M_copy4	H	67	744	810		
ND2	H	1029	811	1839	GTG	TAA
trnW	H	68	1838	1905		
trnC	L	62	1898	1959		
trnY	L	67	1962	2028		
COX1	H	1534	2027	3560	CCG	T
trnL	H	66	3561	3626		
COX2	H	688	3631	4318	ATG	T
trnK	H	71	4319	4389		
trnD	H	66	4389	4454		
ATP8	H	159	4455	4613	ATT	TAA
ATP6	H	675	4610	5284	ATA	TAA
COX3	H	789	5284	6072	ATG	TAA
trnG	H	64	6078	6141		
ND3	H	348	6148	6495	ATT	TAA
trnA	H	64	6499	6562		
trnR	H	64	6562	6625		
trnN	H	65	6625	6689		
trnS	H	67	6690	6756		
trnE	H	64	6758	6821		
trnF	L	64	6820	6883		
ND5	L	1735	6884	8618	ATG	T
trnH	L	63	8620	8682		
ND4	L	1347	8683	10029	ATG	TAG
ND4L	L	297	10023	10319	ATG	TAA
trnT	H	64	10322	10385		
trnP	L	65	10386	10450		
ND6	H	510	10471	10980	ATT	TAA
Cyt b	H	1137	10980	12116	ATG	TAG
S_copy2	H	70	12115	12184		
ND1	L	945	12204	13148	ATG	TAA
L_copy2	L	67	13150	13216		
rrnL	L	1298	13217	14514		
trnV	L	71	14515	14585		
rrnS	L	790	14586	15375		

D. *Ameletus cedrensis* JLFY13

Gene	Strand	Size	Position		Codon	
			From	To	Start	Stop
trnI	H	67	1	67		
trnQ	L	69	65	133		
trnM	H	65	133	197		
M_copy2	H	65	253	317		
ND2	H	1035	318	1352	GTG	TAA
trnW	H	68	1351	1418		
trnC	L	63	1411	1473		
trnY	L	65	1474	1538		
COX1	H	1540	1531	3070	ATT	T
trnL	H	67	3071	3137		
COX2	H	688	3145	3832	ATG	T
trnK	H	70	3833	3902		
trnD	H	66	3902	3967		
ATP8	H	159	3968	4126	ATT	TAA
ATP6	H	675	4123	4797	ATA	TAA
COX3	H	789	4797	5585	ATG	TAA
trnG	H	62	5587	5648		
ND3	H	354	5649	6002	ATG	TAG
trnA	H	64	6001	6064		
trnR	H	63	6064	6126		
trnN	H	63	6126	6188		
trnS	H	67	6188	6254		
trnE	H	64	6255	6318		
trnF	L	64	6317	6380		
ND5	L	1735	6381	8115	GTG	T
trnH	L	64	8116	8179		
ND4	L	1347	8179	9525	ATG	TAG
ND4L	L	297	9519	9815	ATG	TAA
trnT	H	64	9818	9881		
trnP	L	65	9882	9946		
ND6	H	513	9958	10470	ATA	TAA
Cyt b	H	1137	10470	11606	ATG	TAG
S_copy2	H	70	11605	11674		
ND1	L	951	11679	12629	GTG	TAA
L_copy2	L	65	12630	12694		
rrnL	L	1286	12695	13980		
trnV	L	70	13981	14050		
rrnS	L	785	14051	14835		

E. *Ameletus montanus* NLTYT3

Gene	Strand	Size	Position		Codon	
			From	To	Start	Stop
trnI	H	67	1	67		
trnQ	L	69	65	133		
trnM	H	66	133	198		
M_copy2	H	66	254	319		
ND2	H	1035	320	1354	ATG	TAA
trnW	H	68	1353	1420		
trnC	L	63	1413	1475		
trnY	L	66	1476	1541		
COX1	H	1540	1534	3073	ATT	T
trnL	H	66	3074	3139		
COX2	H	688	3144	3831	ATG	T
trnK	H	70	3832	3901		
trnD	H	66	3901	3966		
ATP8	H	159	3967	4125	ATC	TAA
ATP6	H	675	4122	4796	ATA	TAA
COX3	H	789	4796	5584	ATG	TAA
trnG	H	62	5585	5646		
ND3	H	354	5647	6000	ATG	TAG
trnA	H	64	5999	6062		
trnR	H	63	6062	6124		
trnN	H	63	6124	6186		
trnS	H	67	6186	6252		
trnE	H	64	6253	6316		
trnF	L	64	6315	6378		
ND5	L	1735	6379	8113	GTG	T
trnH	L	63	8114	8176		
ND4	L	1347	8176	9522	ATG	TAG
ND4L	L	297	9516	9812	ATG	TAA
trnT	H	64	9815	9878		
trnP	L	65	9879	9943		
ND6	H	519	9949	10467	ATT	TAA
Cyt b	H	1137	10467	11603	ATG	TAG
S_copy2	H	70	11602	11671		
ND1	L	945	11682	12626	GTG	TAA
L_copy2	L	67	12627	12693		
rrnL	L	1288	12694	13981		
trnV	L	70	13982	14051		
rrnS	L	787	14052	14838		

F. *Ameletus* sp. LNFSFY1

**Gene**	**Strand**	**Size**	**Position**		**Codon**	
			**From**	**To**	**Start**	**Stop**
trnI	H	67	1	67		
trnQ	L	69	65	133		
trnM	H	66	133	198		
M_copy2	H	66	253	318		
ND2	H	1029	319	1347	ATT	TAA
trnW	H	68	1346	1413		
trnC	L	62	1406	1467		
trnY	L	63	1468	1530		
COX1	H	1540	1523	3062	ATT	T
trnL	H	67	3063	3129		
COX2	H	688	3134	3821	ATG	T
trnK	H	70	3822	3891		
trnD	H	66	3891	3956		
ATP8	H	159	3957	4115	ATC	TAA
ATP6	H	675	4112	4786	ATA	TAA
COX3	H	789	4786	5574	ATG	TAG
trnG	H	62	5576	5637		
ND3	H	354	5638	5991	ATA	TAG
trnA	H	64	5990	6053		
trnR	H	63	6053	6115		
trnN	H	62	6116	6177		
trnS	H	67	6177	6243		
trnE	H	63	6244	6306		
trnF	L	64	6305	6368		
ND5	L	1723	6369	8091	ATT	T
trnH	L	62	8104	8165		
ND4	L	1347	8165	9511	GTG	TAA
ND4L	L	297	9505	9801	ATG	TAA
trnT	H	63	9804	9866		
trnP	L	65	9867	9931		
ND6	H	519	9937	10455	ATT	TAA
Cyt b	H	1137	10455	11591	ATG	TAG
S_copy2	H	70	11590	11659		
ND1	L	951	11664	12614	GTG	TAA
L_copy2	L	68	12614	12681		
rrnL	L	1283	12682	13964		
trnV	L	70	13965	14034		
rrnS	L	775	14035	14809		

G. *Ameletus* sp. BZD6

Gene	Strand	Size	Position		Codon	
			From	To	Start	Stop
trnI	H	67	1	67		
trnQ	L	69	65	133		
trnM	H	65	133	197		
M_copy2	H	65	251	315		
ND2	H	1035	316	1350	GTG	TAA
trnW	H	68	1349	1416		
trnC	L	63	1409	1471		
trnY	L	66	1472	1537		
COX1	H	1540	1530	3069	ATT	T
trnL	H	66	3070	3135		
COX2	H	688	3140	3827	ATG	T
trnK	H	70	3828	3897		
trnD	H	66	3897	3962		
ATP8	H	159	3963	4121	ATC	TAA
ATP6	H	675	4118	4792	ATA	TAA
COX3	H	789	4792	5580	ATG	TAA
trnG	H	62	5581	5642		
ND3	H	354	5643	5996	ATG	TAG
trnA	H	64	5995	6058		
trnR	H	63	6058	6120		
trnN	H	63	6120	6182		
trnS	H	67	6182	6248		
trnE	H	64	6249	6312		
trnF	L	64	6311	6374		
ND5	L	1735	6375	8109	GTG	T
trnH	L	63	8110	8172		
ND4	L	1347	8172	9518	ATG	TAG
ND4L	L	297	9512	9808	ATG	TAA
trnT	H	64	9811	9874		
trnP	L	65	9875	9939		
ND6	H	519	9945	10463	ATT	TAA
Cyt b	H	1137	10463	11599	ATG	TAG
S_copy2	H	70	11598	11667		
ND1	L	945	11678	12622	GTG	TAA
L_copy2	L	67	12623	12689		
rrnL	L	1288	12690	13977		
trnV	L	70	13978	14047		
rrnS	L	785	14048	14832		

H. *Isonychia taishunensis* DXY3

Gene	Strand	Size	Position		Codon	
			From	To	Start	Stop
trnI	H	64	1	64		
trnQ	L	69	62	130		
trnM	H	66	130	195		
ND2	H	1026	196	1221	GTG	TAA
trnW	H	68	1220	1287		
trnC	L	62	1280	1341		
trnY	L	65	1342	1406		
COX1	H	1534	1405	2938	CCG	T
trnL	H	67	2939	3005		
COX2	H	688	3010	3697	ATG	T
trnK	H	69	3698	3766		
trnD	H	67	3766	3832		
ATP8	H	159	3833	3991	ATC	TAA
ATP6	H	675	3988	4662	ATA	TAA
COX3	H	789	4662	5450	ATG	TAA
trnG	H	63	5452	5514		
ND3	H	354	5515	5868	ATT	TAG
trnA	H	64	5867	5930		
trnR	H	63	5930	5992		
trnN	H	62	5993	6054		
trnS	H	66	6054	6119		
trnE	H	64	6120	6183		
trnF	L	65	6182	6246		
ND5	L	1735	6247	7981	GTG	T
trnH	L	63	7982	8044		
ND4	L	1345	8045	9389	ATG	T
ND4L	L	297	9383	9679	ATG	TAA
trnT	H	63	9734	9796		
trnP	L	65	9797	9861		
ND6	H	507	9873	10379	ATA	TAA
Cyt b	H	1135	10379	11513	ATG	T
S_copy2	H	70	11514	11583		
ND1	L	963	11576	12538	ATG	TAG
L_copy2	L	66	12540	12605		
rrnL	L	1285	12606	13890		
trnV	L	71	13891	13961		
rrnS	L	790	13962	14751		

I. *Isonychia valida* JLH11

Gene	Strand	Size	Position		Codon	
			From	To	Start	Stop
trnI	H	64	1	64		
trnQ	L	69	62	130		
trnM	H	66	131	196		
ND2	H	1026	197	1222	GTG	TAA
trnW	H	68	1221	1288		
trnC	L	62	1281	1342		
trnY	L	64	1343	1406		
COX1	H	1534	1405	2938	CCG	T
trnL	H	66	2939	3004		
COX2	H	688	3009	3696	ATG	T
trnK	H	68	3697	3764		
trnD	H	66	3765	3830		
ATP8	H	159	3831	3989	ATT	TAA
ATP6	H	678	3983	4660	GTG	TAA
COX3	H	789	4660	5448	ATG	TAA
trnG	H	63	5452	5514		
ND3	H	354	5515	5868	ATC	TAA
trnA	H	64	5868	5931		
trnR	H	63	5931	5993		
trnN	H	61	5994	6054		
trnS	H	66	6055	6120		
trnE	H	64	6121	6184		
trnF	L	64	6183	6246		
ND5	L	1738	6244	7981	GTG	T
trnH	L	65	7982	8046		
ND4	L	1345	8047	9391	ATG	T
ND4L	L	297	9385	9681	ATG	TAA
trnT	H	64	9729	9792		
trnP	L	65	9793	9857		
ND6	H	510	9869	10378	ATA	TAA
Cyt b	H	1135	10378	11512	ATG	T
S_copy2	H	70	11513	11582		
ND1	L	949	11589	12537	ATG	T
L_copy2	L	66	12539	12604		
rrnL	L	1283	12605	13887		
trnV	L	71	13888	13958		
rrnS	L	796	13959	14754		

J. *Isonychia bicolor* 03FY38

Gene	Strand	Size	Position		Codon	
			From	To	Start	Stop
trnI	H	64	1	64		
trnQ	L	69	62	130		
trnM	H	65	130	194		
ND2	H	1023	195	1217	GTG	TAA
trnW	H	68	1216	1283		
trnC	L	62	1276	1337		
trnY	L	65	1338	1402		
COX1	H	1534	1401	2934	CCG	T
trnL	H	66	2935	3000		
COX2	H	688	3005	3692	ATG	T
trnK	H	69	3693	3761		
trnD	H	66	3761	3826		
ATP8	H	159	3827	3985	ATT	TAA
ATP6	H	675	3982	4656	ATA	TAA
COX3	H	789	4656	5444	ATG	TAA
trnG	H	64	5446	5509		
ND3	H	354	5510	5863	ATG	TAG
trnA	H	64	5862	5925		
trnR	H	63	5925	5987		
trnN	H	63	5988	6050		
trnS	H	66	6050	6115		
trnE	H	64	6116	6179		
trnF	L	64	6178	6241		
ND5	L	1735	6242	7976	GTG	T
trnH	L	63	7977	8039		
ND4	L	1345	8040	9384	ATG	T
ND4L	L	297	9378	9674	ATG	TAA
trnT	H	64	9727	9790		
trnP	L	65	9791	9855		
ND6	H	501	9870	10370	ATA	TAA
Cyt b	H	1135	10370	11504	ATG	T
S_copy2	H	70	11505	11574		
ND1	L	939	11591	12529	ATG	TAG
L_copy2	L	66	12531	12596		
rrnL	L	1283	12597	13879		
trnV	L	71	13880	13950		
rrnS	L	803	13951	14753		

K. *Isonychia kiangsinensis* 02JXDF

Gene	Strand	Size	Position		Codon	
			From	To	Start	Stop
trnI	H	64	1	64		
trnQ	L	69	62	130		
trnM	H	66	131	196		
ND2	H	1020	197	1216	GTG	TAA
trnW	H	68	1215	1282		
trnC	L	63	1275	1337		
trnY	L	64	1339	1402		
COX1	H	1540	1395	2934	ATC	T
trnL	H	65	2935	2999		
COX2	H	688	3004	3691	ATG	T
trnK	H	69	3692	3760		
trnD	H	66	3760	3825		
ATP8	H	159	3826	3984	ATC	TAA
ATP6	H	675	3981	4655	ATA	TAA
COX3	H	789	4655	5443	ATG	TAA
trnG	H	63	5446	5508		
ND3	H	354	5509	5862	ATT	TAG
trnA	H	64	5861	5924		
trnR	H	63	5924	5986		
trnN	H	62	5987	6048		
trnS	H	65	6048	6112		
trnE	H	63	6113	6175		
trnF	L	64	6174	6237		
ND5	L	1735	6238	7972	GTG	T
trnH	L	63	7973	8035		
ND4	L	1345	8036	9380	ATG	T
ND4L	L	297	9374	9670	ATG	TAA
trnT	H	63	9718	9780		
trnP	L	65	9781	9845		
ND6	H	507	9851	10357	ATA	TAA
Cyt b	H	1135	10357	11491	ATG	T
S_copy2	H	70	11492	11561		
ND1	L	963	11554	12516	ATG	TAG
L_copy2	L	65	12518	12582		
rrnL	L	1274	12583	13856		
trnV	L	70	13858	13927		
rrnS	L	780	13928	14707		

L. *Isonychia kiangsinensis* 02WZ02

Gene	Strand	Size	Position		Codon	
			From	To	Start	Stop
trnI	H	64	1	64		
trnQ	L	69	62	130		
trnM	H	66	131	196		
ND2	H	1020	197	1216	GTG	TAA
trnW	H	68	1215	1282		
trnC	L	63	1275	1337		
trnY	L	64	1339	1402		
COX1	H	1540	1395	2934	ATC	T
trnL	H	65	2935	2999		
COX2	H	688	3004	3691	ATG	T
trnK	H	69	3692	3760		
trnD	H	66	3760	3825		
ATP8	H	159	3826	3984	ATC	TAA
ATP6	H	675	3981	4655	ATA	TAA
COX3	H	789	4655	5443	ATG	TAA
trnG	H	63	5446	5508		
ND3	H	354	5509	5862	ATT	TAG
trnA	H	64	5861	5924		
trnR	H	63	5924	5986		
trnN	H	62	5987	6048		
trnS	H	65	6048	6112		
trnE	H	63	6113	6175		
trnF	L	64	6174	6237		
ND5	L	1735	6238	7972	GTG	T
trnH	L	63	7973	8035		
ND4	L	1345	8036	9380	ATG	T
ND4L	L	297	9374	9670	ATG	TAA
trnT	H	63	9718	9780		
trnP	L	65	9781	9845		
ND6	H	507	9851	10357	ATA	TAA
Cyt b	H	1135	10357	11491	ATG	T
S_copy2	H	70	11492	11561		
ND1	L	963	11554	12516	ATG	TAG
L_copy2	L	65	12518	12582		
rrnL	L	1276	12583	13858		
trnV	L	70	13859	13928		
rrnS	L	783	13929	14711		

M. *Isonychia kiangsinensis* 02WZ04

Gene	Strand	Size	Position		Codon	
			From	To	Start	Stop
trnI	H	64	1	64		
trnQ	L	69	62	130		
trnM	H	66	131	196		
ND2	H	1020	197	1216	GTG	TAA
trnW	H	68	1215	1282		
trnC	L	63	1275	1337		
trnY	L	64	1339	1402		
COX1	H	1540	1395	2934	ATC	T
trnL	H	65	2935	2999		
COX2	H	688	3004	3691	ATG	T
trnK	H	69	3692	3760		
trnD	H	66	3760	3825		
ATP8	H	159	3826	3984	ATC	TAA
ATP6	H	675	3981	4655	ATA	TAA
COX3	H	789	4655	5443	ATG	TAA
trnG	H	63	5446	5508		
ND3	H	354	5509	5862	ATT	TAG
trnA	H	64	5861	5924		
trnR	H	63	5924	5986		
trnN	H	62	5987	6048		
trnS	H	65	6048	6112		
trnE	H	63	6113	6175		
trnF	L	64	6174	6237		
ND5	L	1735	6238	7972	GTG	T
trnH	L	63	7973	8035		
ND4	L	1345	8036	9380	ATG	T
ND4L	L	297	9374	9670	ATG	TAA
trnT	H	63	9718	9780		
trnP	L	65	9781	9845		
ND6	H	507	9851	10357	ATA	TAA
Cyt b	H	1135	10357	11491	ATG	T
S_copy2	H	70	11492	11561		
ND1	L	963	11554	12516	ATG	TAG
L_copy2	L	65	12518	12582		
rrnL	L	1276	12583	13858		
trnV	L	70	13859	13928		
rrnS	L	783	13929	14711		

N. *Isonychia* sp. JLS1

Gene	Strand	Size	Position		Codon	
			From	To	Start	Stop
trnI	H	64	1	64		
trnQ	L	69	62	130		
trnM	H	68	131	198		
ND2	H	1026	199	1224	ATG	TAA
trnW	H	68	1223	1290		
trnC	L	62	1283	1344		
trnY	L	65	1345	1409		
COX1	H	1534	1408	2941	CCG	T
trnL	H	66	2942	3007		
COX2	H	688	3012	3699	ATG	T
trnK	H	69	3700	3768		
trnD	H	68	3768	3835		
ATP8	H	159	3836	3994	ATT	TAA
ATP6	H	675	3991	4665	ATA	TAA
COX3	H	789	4665	5453	ATG	TAG
trnG	H	63	5455	5517		
ND3	H	354	5518	5871	ATC	TAG
trnA	H	64	5870	5933		
trnR	H	63	5933	5995		
trnN	H	62	5996	6057		
trnS	H	65	6057	6121		
trnE	H	64	6122	6185		
trnF	L	64	6184	6247		
ND5	L	1735	6248	7982	ATG	T
trnH	L	63	7983	8045		
ND4	L	1345	8046	9390	GTG	T
ND4L	L	297	9384	9680	ATG	TAA
trnT	H	63	9737	9799		
trnP	L	65	9800	9864		
ND6	H	507	9876	10382	ATA	TAA
Cyt b	H	1135	10382	11516	ATG	T
S_copy2	H	70	11517	11586		
ND1	L	963	11579	12541	ATG	TAG
L_copy2	L	66	12543	12608		
rrnL	L	1288	12609	13896		
trnV	L	69	13897	13965		
rrnS	L	789	13966	14754		

O. *Isonychia* sp. 02WZ09

Gene	Strand	Size	Position		Codon	
			From	To	Start	Stop
trnI	H	65	1	65		
trnQ	L	69	63	131		
trnM	H	66	131	196		
ND2	H	1026	197	1222	ATG	TAA
trnW	H	68	1221	1288		
trnC	L	62	1281	1342		
trnY	L	65	1343	1407		
COX1	H	1534	1406	2939	CCG	T
trnL	H	67	2940	3006		
COX2	H	688	3011	3698	ATG	T
trnK	H	69	3699	3767		
trnD	H	67	3767	3833		
ATP8	H	159	3834	3992	ATC	TAA
ATP6	H	675	3989	4663	ATA	TAA
COX3	H	789	4663	5451	ATG	TAA
trnG	H	63	5453	5515		
ND3	H	354	5516	5869	ATT	TAG
trnA	H	64	5868	5931		
trnR	H	63	5931	5993		
trnN	H	62	5994	6055		
trnS	H	66	6055	6120		
trnE	H	63	6121	6183		
trnF	L	65	6182	6246		
ND5	L	1735	6247	7981	GTG	T
trnH	L	63	7982	8044		
ND4	L	1345	8045	9389	ATG	T
ND4L	L	297	9383	9679	ATG	TAA
trnT	H	63	9734	9796		
trnP	L	65	9797	9861		
ND6	H	507	9873	10379	ATA	TAA
Cyt b	H	1135	10379	11513	ATG	T
S_copy2	H	70	11514	11583		
ND1	L	963	11576	12538	ATG	TAG
L_copy2	L	66	12540	12605		
rrnL	L	1284	12606	13889		
trnV	L	71	13891	13961		
rrnS	L	787	13962	14748		

P. *Isonychia* sp. 9GZST

Gene	Strand	Size	Position		Codon	
			From	To	Start	Stop
trnI	H	64	1	64		
trnQ	L	69	62	130		
trnM	H	66	131	196		
ND2	H	1026	197	1222	GTG	TAA
trnW	H	68	1221	1288		
trnC	L	63	1281	1343		
trnY	L	65	1344	1408		
COX1	H	1534	1408	2941	CCG	T
trnL	H	65	2942	3006		
COX2	H	688	3011	3698	ATG	T
trnK	H	69	3699	3767		
trnD	H	66	3767	3832		
ATP8	H	159	3833	3991	ATT	TAA
ATP6	H	675	3988	4662	ATA	TAA
COX3	H	789	4662	5450	ATG	TAA
trnG	H	63	5454	5516		
ND3	H	354	5517	5870	ATC	TAG
trnA	H	64	5869	5932		
trnR	H	63	5932	5994		
trnN	H	62	5995	6056		
trnS	H	65	6056	6120		
trnE	H	64	6121	6184		
trnF	L	64	6183	6246		
ND5	L	1713	6249	7961	ATT	TAG
trnH	L	63	7980	8042		
ND4	L	1345	8043	9387	ATG	T
ND4L	L	297	9381	9677	ATG	TAA
trnT	H	62	9728	9789		
trnP	L	65	9790	9854		
ND6	H	513	9860	10372	ATA	TAA
Cyt b	H	1135	10372	11506	ATG	T
S_copy2	H	70	11507	11576		
ND1	L	963	11569	12531	ATG	TAG
L_copy2	L	65	12533	12597		
rrnL	L	1275	12598	13872		
trnV	L	70	13873	13942		
rrnS	L	778	13943	14720		
