# Supplementary material for: Comparative Mitogenomics Reveals Gene Rearrangement and Phylogenetic Relationships in Siphlonuroidea (Insecta: Ephemeroptera)
Source: Insects. 2026 Jul 11;17(7):718. doi: 10.3390/insects17070718 (PMC13410250; doi:10.3390/insects17070718)
Supplement: Supplementary file 1 [file insects-17-00718-s001.zip › Table S6.pdf]

Table S6. The divergence-time of each family.

Family	Mean divergence-time (Ma)	95% HPD Range (Ma)
Siphluriscidae	211.61	184.65 - 242.74
Ameletidae & Siphlonuridae	174.71	162.45 - 190.03
Isonychiidae & Heptageniidae	136.81	76.27 - 188.72
Leptophlebiidae	176.45	149.91 - 204.87
Teloganodidae & Baetidae	140.09	110.10 - 170.60
Vietnamellidae & Ephemerellidae	136.03	111.71 - 164.05
Euthyplociidae	113.30	74.91 - 158.28
Neoephemeridae & Caenidae	93.72	62.14 - 133.70
Potamanthidae	104.53	63.92 - 154.98
Behningiidae	83.91	43.86 - 126.78
Ephemeridae	72.79	38.57 – 112.26
Palingeniidae & Polymitarcyidae	53.28	22.94 - 92.59
