# Supplementary material for: Comparative Mitogenomics Reveals Gene Rearrangement and Phylogenetic Relationships in Siphlonuroidea (Insecta: Ephemeroptera)
Source: Insects. 2026 Jul 11;17(7):718. doi: 10.3390/insects17070718 (PMC13410250; doi:10.3390/insects17070718)
Supplement: Supplementary file 1 [file Figure S5.pdf › Figure S5.pdf]

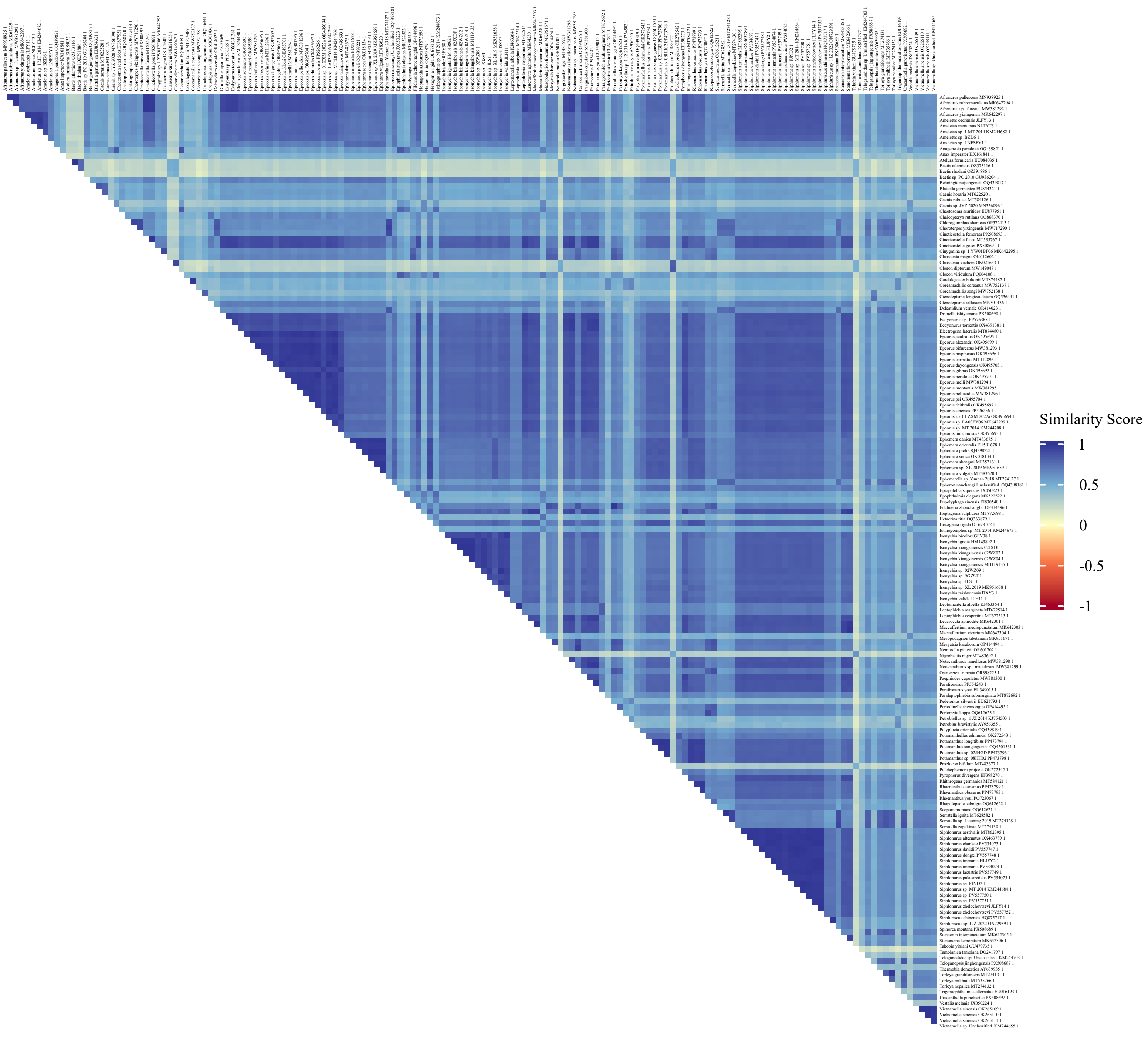

Figure S5. Heterogeneity test of the concatenated dataset consisting of the first and second codon positions (PCG12) of protein-coding genes from 164 insect mitochondrial genomes.
